# HCV and flaviviruses hijack cellular mechanisms for nuclear STAT2 degradation: Up-regulation of PDLIM2 suppresses the innate immune response

**DOI:** 10.1371/journal.ppat.1007949

**Published:** 2019-08-02

**Authors:** Michael A. Joyce, Karyn M. Berry-Wynne, Theodore dos Santos, William R. Addison, Nicola McFarlane, Tom Hobman, D. Lorne Tyrrell

**Affiliations:** 1 Department of Medical Microbiology and Immunology, University of Alberta, Edmonton, Alberta, Canada; 2 Li Ka Shing Institute of Virology, University of Alberta, Edmonton, Alberta, Canada; 3 Department of Cell Biology, University of Alberta, Edmonton, Alberta, Canada; The University of Chicago, UNITED STATES

## Abstract

Host encounters with viruses lead to an innate immune response that must be rapid and broadly targeted but also tightly regulated to avoid the detrimental effects of unregulated interferon expression. Viral stimulation of host negative regulatory mechanisms is an alternate method of suppressing the host innate immune response. We examined three key mediators of the innate immune response: NF-KB, STAT1 and STAT2 during HCV infection in order to investigate the paradoxical induction of an innate immune response by HCV despite a multitude of mechanisms combating the host response. During infection, we find that all three are repressed only in HCV infected cells but not in uninfected bystander cells, both *in vivo* in chimeric mouse livers and in cultured Huh7.5 cells after IFNα treatment. We show here that HCV and Flaviviruses suppress the innate immune response by upregulation of PDLIM2, independent of the host interferon response. We show PDLIM2 is an E3 ubiquitin ligase that also acts to stimulate nuclear degradation of STAT2. Interferon dependent relocalization of STAT1/2 to the nucleus leads to PDLIM2 ubiquitination of STAT2 but not STAT1 and the proteasome-dependent degradation of STAT2, predominantly within the nucleus. CRISPR/Cas9 knockout of PDLIM2 results in increased levels of STAT2 following IFNα treatment, retention of STAT2 within the nucleus of HCV infected cells after IFNα stimulation, increased interferon response, and increased resistance to infection by several flaviviruses, indicating that PDLIM2 is a global regulator of the interferon response.

## Author summary

The response of cells to an invading pathogen must be swift and well controlled because of the detrimental effects of chronic inflammation. However, viruses often hijack host control mechanisms. HCV and flaviviruses are known to suppress the innate immune response in cells by a variety of mechanisms. This study clarifies and expands a specific cellular mechanism for global control of the antiviral response after the induction of interferon expression. It shows how several viruses hijack this control mechanism to suppress the innate interferon response.

## Introduction

Host cells sense the presence of an infecting virus using pathogen pattern recognition receptors which initiate signaling pathways that converge on the activation of NF-κB and the IRF family of transcription factors leading to the production and secretion of cytokines, type I (IFNα and IFNβ) and type III (IFN-λ) interferons [1–5]. Binding of type I and type III IFN to receptors on the infected cell and neighbouring cells leads to nuclear translocation of STAT1/STAT2/IRF9 (ISGF3) [6] and the transcription of numerous IFN stimulated genes (ISGs) which in turn inhibit viral replication [7–9]. Cooperation between the NF-κB and ISGF3 pathways is required to induce an innate immune response that controls a variety of pathogens [9–12]. However, because of the wide variety of biologic functions regulated during the innate immune response, continuous induction of the anti-viral state is detrimental to normal cell function [13, 14], and fine control of the antiviral response is required. Host cells have developed a multitude of mechanisms to shut down the interferon response and viruses have evolved mechanisms to prematurely activate these shut down pathways to enhance their own replication [15].

Canonical interferon signaling initiated by IFN-α or IFN-λ induces dimerization of their receptors, IFNAR1 and IFNAR2 or IFNLR1 and IL-10R2 respectively, activating the associated protein kinases janus kinase1 (JAK1) and tyrosine kinase2 (TYK2). The major substrates of these kinases are signal transducer and activator of transcription 1 and 2 (STAT1 and STAT2). Phosphorylated STAT1 and STAT2 bind IRF9 to form the transcription factor ISGF3, which migrates to the nucleus to induce ISG transcription [13]. However, it has also been shown that STAT2 can homodimerize and stimulate ISG expression independently [16], and while STAT1 is essential for IFN-γ and IFN-λ signaling, STAT2 is essential for both IFN-λ and IFN-α signaling in Huh7.5 cells [17]. In addition, unlike STAT1 knockdown, STAT2 knockdown resulted in elevation of HCV levels in stem cell derived hepatocytes [18]. Control of STAT signaling involves numerous post translational modifications: acetylation [19], methylation [20, 21], and ISGylation [22, 23] promote signaling, while dephosphorylation [24] or sumoylation [25] inhibit it. Nuclear ubiquitination of STAT followed by degradation by the proteasome is another mechanism to shut down STAT mediated transcription [26–28].

Ubiquitination of key signaling proteins and their subsequent degradation in the proteasome has emerged as a method for shutting down a variety of processes including the innate immune response [29–31]. Ubiquitin E3 ligases are the elements of the ubiquitination pathway responsible for substrate specificity. PDLIM2 is one such ubiquitin E3 ligase. Its known targets include NF-κB [32, 33], as well as STAT1, STAT3, and STAT4 in mouse cells or when overexpressed from plasmids [28, 34, 35]. In the case of NF-κB it was shown that the active form of the transcription factor is targeted for degradation in insoluble proteasome complexes within the nucleus.

It has been shown that HCV infected patients have an ongoing innate interferon response in their livers and that a very similar response develops in the chimeric livers of HCV infected SCID/Alb-uPA mice in the absence of an adaptive immune system [36–40]. The host interferon response persists despite a variety of mechanisms that HCV uses to inhibit the interferon response [41]. Studies in cell culture revealed RIGI and downstream IRF3 activation occurs early in infection and subsequently controlled by HCV NS3/4A later in infection [42]. Consistently, studies of patient biopsies revealed that the ISG response originated in HCV infected cells and was also high in neighboring bystander cells [43]. In mice where HCV was expressed in liver cells, the STAT response was suppressed. When overexpressed both HCV core and NS5a reduced STAT1 phosphorylation and interferon signaling [44, 45]. HCV core also stimulates protein phosphatase 2A to inhibit IFN-α signaling but this can be overcome by high concentrations of IFN-α [46, 47]. Surprisingly, STAT1 was not essential for inhibition of HCV by IFN-α [17]. We previously examined NF-κB protein levels [48] in the chimeric livers of HCV infected SCID/Alb-uPA mice and found that NF-kB levels were decreased in HCV infected cells but elevated in surrounding uninfected human hepatocytes. This implied that HCV can blunt innate immunity in infected cells and was the first indication that uninfected bystander cells, not infected cells, were responsible for the global interferon response seen *in vivo* [48].

Here we extend our studies to the mechanisms by which HCV and flaviviruses limit innate immunity. In these studies we focused on the effects of viral infection on the levels and cellular location of two key signal transduction molecules mentioned above, STAT1 and STAT2. During HCV infection of chimeric mouse livers, HCV infected cells have reduced levels of STAT1 and STAT2 compared to bystander cells, and during interferon treatment HCV infected Huh7.5 cells have reduced levels of STAT1 and STAT2 compared to bystander cells. The reduction in levels of nuclear of STAT2, but not STAT1, is blocked by the proteasome inhibitor MG132 implicating the ubiquitination pathway in this process. Infection by HCV and flaviviruses strongly induces transcription of the ubiquitin E3 ligase PDLIM2. Knockout of the PDLIM2 gene results in increased levels of STAT2 after IFNα treatment, the retention of STAT2 in the nucleus of HCV infected cells, a decrease in viral replication, and a more robust interferon response.

## Results

### HCV infected cells contain decreased levels of STAT1, STAT2, and NF-κB during an ongoing interferon response

**Fig 1:**
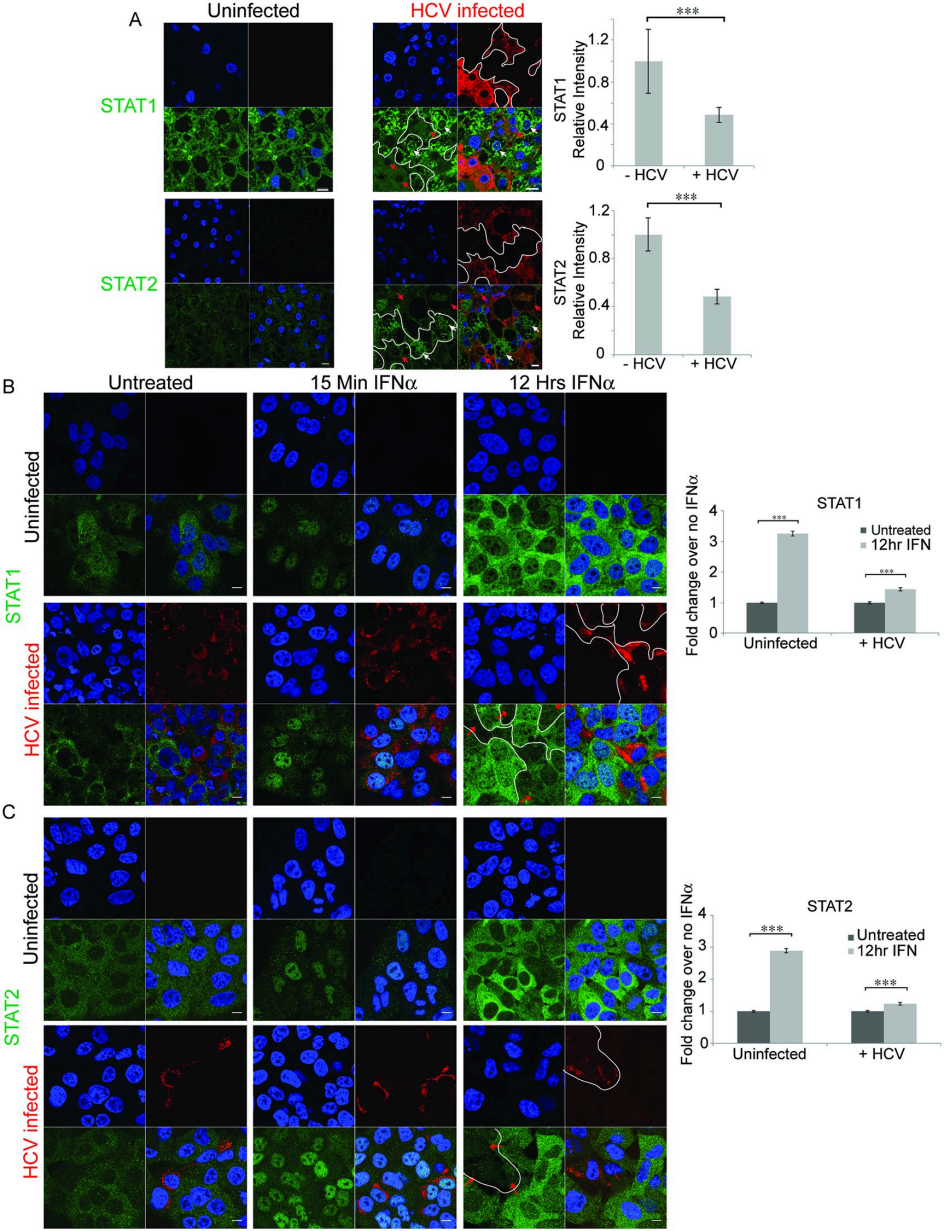
Confocal microscopy reveals lower STAT1 and STAT2 levels in HCV infected cells both *in vivo*, in chimeric mouse livers, and *in vitro*, in Huh7.5 cells. Confocal microscopy was performed on A) liver sections or B) and C) Huh7.5 cells either uninfected or infected with HCV. A) SCID/Alb-uPA mice transplanted with human hepatocytes were infected and liver sections were stained using antibodies directed against HCV (red) and antibodies specific for either STAT1 or STAT2 (green). Isotype controls are shown in S1 Fig. The amount of STAT1 or STAT2 was quantified in cells that stained positive or negative for HCV within an infected liver. For STAT1, 58 infected and 79 uninfected cells were analyzed from 10 fields. For STAT2, 42 infected and 74 uninfected cells were analyzed from 4 fields using Metamorph software. To compare cells among several infected fields the average green (STAT) fluorescence of uninfected cells from a single field was arbitrarily set to 1 and the infected cells in that field were scaled appropriately. In B) and C), confocal microscopy was performed on uninfected or HCV infected Huh7.5 cells that were either left untreated or treated with IFNα2 for the indicated times prior to fixation. Cells were stained using antibodies directed against HCV (Red) and antibodies specific for either B) STAT1 or C) STAT2 (green). For quantification, at least 511 cells in multiple fields for each treatment were quantified. Nuclei were stained with DAPI (blue). The scale bars are 10μm. For reference, white borders were drawn around infected cells in the red channel and copied into the green channel; red arrows mark some HCV infected cells, and white arrows mark some uninfected cells in the last panel. Unpaired t-tests were used to determine significance, and *** denotes p<0.001.

We have previously shown that the interferon response to HCV in chimeric livers of SCID/Alb-uPA mice was similar to that found in liver biopsies of HCV infected patients [40]. We, and others, have shown that despite having several known mechanisms to combat the host interferon response, HCV infection still induces numerous ISGs [49–53]. To address this apparent paradox, we examined how the levels and locations of key mediators of the innate immune response, STAT1 and STAT2 and NF-κB, changed in response to HCV infection in the livers of chimeric mice using confocal microscopy. Comparison of immunostained liver sections from uninfected mice to those from infected mice showed that the overall levels of STAT1, STAT2, and NF-κB trended higher in HCV infected mice (Figs 1A, S1 and S2). Comparison of STAT1 and STAT2 protein levels in uninfected livers and infected livers using confocal microscopy revealed that STAT1 levels increased 1.7±1.3 fold in infected livers while STAT2 increased 1.5±0.45 fold in infected livers. While this was not significant it was consistent with the known induction of an interferon response by HCV in the tissue [40, 48] as well as Huh7.5 cells (S1 Table). However, within infected livers, when we compared the levels of STAT1 or STAT 2 in HCV infected cells (red arrows) with the levels in uninfected cells (white arrows) we saw reduced levels of STAT1 and STAT2 in the HCV infected cells. Quantification revealed that STAT1 and STAT2 levels in infected cells were approximately half that in uninfected cells within an infected liver (Figs 1A and S1B). The same phenomenon was seen with NF-κB expression in infected chimeric liver tissue (S2 Fig), consistent with our previous results [48]. These results indicate that upregulation of STAT signaling and a concomitant interferon response and induction of ISGs (S1 Table and [40]) occurs in bystander cells but HCV infected cells were able to thwart STAT signaling. This is consistent with an inverse correlation between the ISG, IFITM3 and HCV RNA in individual cells of HCV patient livers isolated using laser dissection microscopy [54], and also consistent with the notion that the stimulus for ISG expression originates in HCV infected cells [43].

To further investigate the mechanism leading to decreased levels of STAT1 and STAT2 in HCV infected cells *in vivo*, we extended our observations to a HCV cell culture system. We confirmed previous findings [55] showing ISG induction during HCV JFH-1 infection of Huh7.5 cells (S1 Table). The mRNA of 37/39 ISGs examined increased their expression at least 2 fold during HCV infection. To examine the effect of HCV infection on STAT protein translocation we treated Huh7.5 cells with exogenous interferon and examined STAT1 and STAT2 levels by confocal microscopy. Cells were treated with interferon for either 15 min or 12h then stained using antibodies specific for STAT1 or STAT2 (green) and HCV core (red-Fig 1B and 1C). Prior to IFNα treatment, both STATs were present at low levels predominantly in the cytoplasm of both uninfected and HCV infected cells; after 15 minutes of IFNα treatment, the majority of STAT1 and STAT2 migrated to the cell nuclei and there was little difference in either protein levels or localization between uninfected and HCV infected cells, indicating that HCV is not blocking nuclear translocation. After 12h of IFNα treatment there was a notable increase in the levels of both STAT proteins. In cells not exposed to HCV both STATs were primarily cytoplasmic after 12h of IFNα treatment. In cells exposed to HCV there was a clear difference between infected cells and uninfected bystander cells. Infected cells are stained red for HCV core protein, marked by red arrows and outlined in white. Quantitation of the STAT levels revealed that infected cells had low overall levels of both STATs, similar to those found in cells not treated with IFNα. However, during interferon treatment uninfected cells had 3 fold higher levels of STAT proteins than HCV infected cells. These features of STAT1 and STAT2 localization resemble that seen in the livers of chimeric mice with chronic HCV infection: low levels of cytoplasmic STATs in infected cells and higher levels of STATs in bystander human hepatocytes, indicating that the global interferon response noted in transcriptomics arises from uninfected bystander cells.

### Degradation of STAT2 but not STAT1 occurs after Interferon-α dependent nuclear re-localization of STAT1 and STAT2

**Fig 2:**
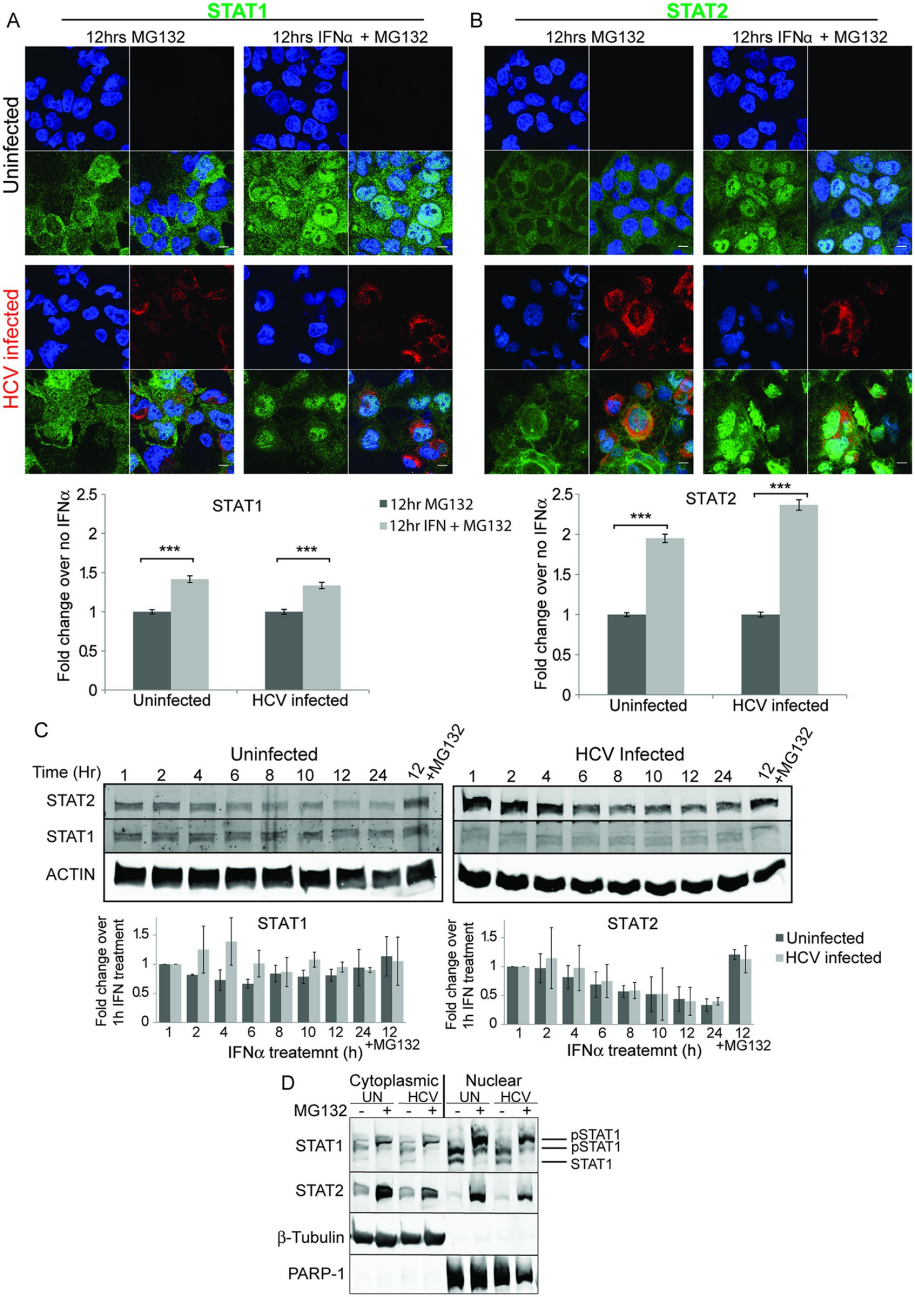
Degradation of STAT2 but not STAT1 after IFNα treatment. Uninfected or HCV infected Huh7.5 cells were either untreated or treated with IFNα2 for 12 h in the presence of the proteasome inhibitor MG132. Confocal microscopy was used to examine either A) STAT1, or B) STAT2 (green). HCV infection was monitored with antibodies specific to HCV core protein (red). Nuclei were stained with DAPI (blue). The scale bars are 10μm. The amount of STAT1 or STAT2 was quantified in at least 594 of each of either uninfected or HCV infected cells. Unpaired t-tests were used to determine significance, and *** denotes p<0.001. C) Uninfected or HCV infected Huh7.5 cells were treated with IFNα2 and the protein synthesis inhibitor cycloheximide for the indicated times. A control, in which proteasomal degradation was inhibited, was additionally treated with MG132. Total cellular protein was separated by SDS PAGE and analyzed by western blotting using antibodies specific for STAT1, STAT2 or actin as a loading control. D) Sub-cellular fractionation. Huh7.5 cells were treated as in C, except following 12 h of IFNα2 and cycloheximide treatment the cells were fractionated into nuclear and cytoplasmic fractions prior to western blot analysis. Western blot analysis for β-tubulin and PARP-1 showed the purity of the two cell fractions and acted as loading controls for proteins in the cytoplasmic and nuclear compartments respectively.

Low levels of STAT1 and STAT2 seen in HCV infected cells may be the result of either a lack of induction or increased degradation. If degradation is involved then STAT protein levels should increase upon treatment with a proteasome inhibitor such as MG132. We included MG132 during 12h IFNα treatment of uninfected and HCV infected cells and then examined STAT1 and STAT2 levels and location (Fig 2A and 2B). Treatment with MG132 alone does not change either localization or amount of STAT1 or STAT2. A small induction of STAT1 was caused by IFNα in the presence of MG132. In contrast to STAT1, inclusion of MG132 during IFNα treatment resulted in significantly increased levels of STAT2 in infected cells indicating that STAT2 is degraded following IFNα treatment. Even more remarkable is the localization of the STAT proteins. Compared to what is seen when cells were treated with IFNα alone (Fig 1B and 1C), treatment with IFNα and MG132 for 12h caused retention of both STAT1 (Fig 2A) and STAT2 (Fig 2B) in the nucleus of infected cells. A similar analysis of the levels of STAT and phospho-STAT was consistent with our confocal analysis (S3 Fig). First, HCV infection led to a decrease in STAT2 but not STAT1 protein levels, indicating HCV infection affects STAT2 levels (S3A Fig). Treatment of cells with IFNα induced phosphorylation of both STAT1 and STAT2, and additional treatment with the proteasome inhibitor MG132 increased the levels of phospho-STAT1 and phospho-STAT2, consistent with nuclear localization. The levels of total STAT1 remained constant after IFNα treatment; however, total STAT2 levels declined during interferon treatment, and this was prevented by MG132. The levels of total STAT2 were also lower in infected and IFNα treated cells (S3B Fig). The levels of HCV core protein did not change significantly during interferon treatment (S3C Fig). Similar experiments monitoring the localization of NF-κB after stimulation with LPS/IL-1β also showed that MG132 treatment led to nuclear retention of NF-κB (S4 Fig) [33, 56].

Since STAT1 and STAT2 protein levels are themselves induced by IFNα, to more clearly examine whether they were degraded during IFNα treatment we performed a time course of IFNα treatment in the presence of the protein synthesis inhibitor cycloheximide (Fig 2C and 2D). Total cell lysates from uninfected or HCV infected Huh7.5 cells were prepared after IFNα treatment, and STAT1 and STAT2 levels were measured by western blot analysis. The levels of STAT1 remained constant over the course of the experiment, and did not change when the proteasome inhibitor MG132 was included during the experiment, indicating STAT1 was not degraded in the absence of new protein synthesis. The levels of STAT2 declined in the presence of IFNα in both uninfected and HCV infected cells. The decrease in STAT2 levels was dependent on the proteasome since it was prevented by MG132 treatment. To examine the sub-cellular compartment where degradation occurred, we fractionated cells into cytoplasmic and nuclear fractions after 12h of IFNα treatment again in the presence of cycloheximide to prevent new protein synthesis (Fig 2D). STAT1 was significantly more abundant in the nucleus than the cytoplasm. Consistent with time course experiments, STAT1 levels did not change upon MG132 treatment. An apparent molecular weight increase was observed in the presence of MG132 which may indicate hyper-phosphorylation STAT1 [57], [20]. Unlike STAT1, when proteasomal degradation was inhibited by MG132, STAT2 levels were elevated. This effect was most dramatic in the nuclear fraction, showing that STAT2 but not STAT1 is degraded predominantly in the nucleus following interferon treatment. Taken together these results may indicate inhibition of STAT2 degradation by MG132 also results in nuclear retention of STAT1, its partner in ISGF3.

### HCV, Dengue virus and Zika virus infection upregulate the E3 ubiquitin ligase PDLIM2

**Fig 3:**
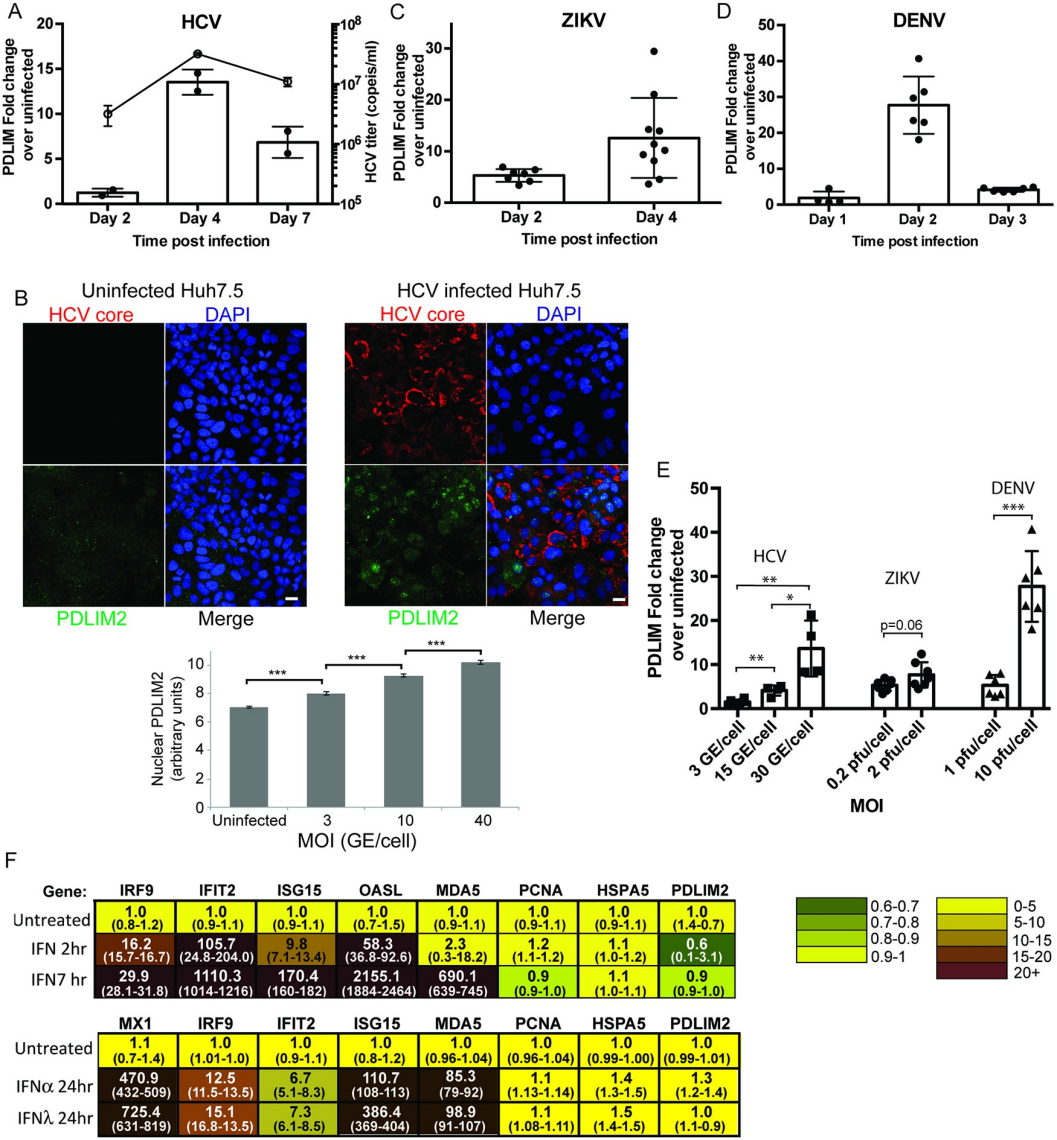
PDLIM2 is induced in virally infected cells but not in cells treated with IFN2α. Huh7.5 cells were infected with either 3 genomes/cell of HCV JFH-1 (A and B) or 0.2 pfu/cell of ZIKV (C), or 10 genomes/cell of DENV (D) and harvested on the days indicated. The levels *PDLIM2* mRNA were quantified by qRT-PCR, normalized to HPRT levels and compared to the amount in uninfected cells. In the case of HCV, cell culture supernatants were also harvested, virus isolated and the HCV viral titers determined by qRT-PCR (shown as the line graph in A). B) Uninfected or HCV infected cells were fixed and stained with antibodies specific for PDLIM2 (green) or HCV core (red) and a minimum of 416 cells at each moi were quantified. Error bars indicate SEM. Unpaired t-tests were used to determine significance, and *** denotes p<0.001. E) Huh7.5 cells were infected with the indicated MOI of HCV, ZIKV, or DENV, and the cells harvested after 4d, 2d, or 2d respectively, and the *PDLIM2* mRNA levels determined by qRT-PCR. Error bars indicate standard deviation. F) Interferon response gene expression (ISG) in Huh7.5 cells treated with IFNα2. Huh7.5 cells were untreated or treated with 1000 IU/mL of IFNα2 for 2h or 7h or 24h, or 100 ng/mL IFN-λ3 for 24h. RNA was isolated and qRT-PCR was performed using a custom TaqMan OpenArray. HPRT was used to normalize mRNA levels and the average of the duplicate untreated sample was used to determine the fold increase after interferon treatment. The range is shown in brackets below the average.

The E3 ubiquitin ligase PDLIM2 has been shown to be one factor capable of shutting down NF-κB, STAT1, STAT3, and STAT4 signaling. It can ubiquitinate these substrates and target them for degradation in the proteasome [28, 32–35, 58]. Since we had originally observed low levels of STAT1, STAT2 and NF-κB in the livers of chimeric mice, we performed similar experiments examining the localization of NF-κB. Stimulation of Huh7.5 cells with IL-1β and LPS led to the nuclear translocation of NF-κB (S4 Fig). HCV infected cells contained less NF-κB than surrounding bystander cells after 7h of LPS/IL-1β stimulation. Taken together these results suggested that a common factor was involved in diminishing STAT and NF-κB activity in HCV infected hepatocytes. We therefore examined the levels of *PDLIM2* mRNA in HCV JFH-1 infected Huh7.5 cells by quantitative RT-PCR (qRT-PCR). In time course experiments we found that *PDLIM2* mRNA levels were 13 fold higher in Huh7.5 cells 4 days after infection, when viral titers peaked, than in uninfected cells (Fig 3A). In dose response experiments, we found *PDLIM2* transcript levels correlated with increasing amounts of HCV JFH-1 virus used to infect Huh7.5 cells (Fig 3E). We also examined PDLIM2 protein levels after infection with HCV (Fig 3B) and found that PDLIM2 increased in the nucleus during infection, consistent with its location and action during NF-KB and HTLV Tax degradation [33, 59]. Interestingly PDLIM2 sequestration in the cytosol by association with the cytoskeleton is associated with increased activity of NF-kB [32, 60], and HCV and flaviviruses are known to disrupt the cytoskeleton [61–65]. Since degradation of STAT2 has been observed in cells infected with other flaviviruses [66–68], we also examined *PDLIM2* expression in Huh7.5 cells infected with Zika virus or Dengue virus (Fig 3C and 3D). Expression of *PDLIM2* mRNA increased 14 fold after Zika virus infection, and was dependent on the amount of initial inoculum (Fig 3C and 3E). Dengue virus infection had the most dramatic effect on *PDLIM2* expression with a 20 fold increase in *PDLIM2* expression soon after infection followed by a rapid decline, again in a MOI dependent manner (Fig 3D and 3E). Representative images showing infection at different MOI with each of HCV, ZIKV, and DENV are shown in S5 Fig. The increase in *PDLIM2* levels in response to a variety of infections implied that *PDLIM2* may be an interferon response gene. To examine whether *PDLIM2* is an ISG, we treated Huh7.5 cells with 1000 IU/mL of IFNα2 for 2h and 7h (Fig 3F). Transcripts for the known ISGs *IRF9*, *IFIT2*, *ISG15*, *OASL*, *and MDA5* increased strongly, as expected. In contrast, *PDLIM2* did not change, nor did the non-ISG’s *PCNA* and *HSPA5* indicating that *PDLIM2* is not an ISG. In separate experiments *PDLIM2* levels also did not change after induction with either IFNα or IFNλ for 24h (Fig 3F). Together, these results indicate viral infection leads to induction of *PDLIM2*, a non-ISG which targets key signal transduction intermediates such as STATs and NF-κB for degradation, and may be a common mechanism whereby flaviviruses inhibit the host interferon response.

### STAT2 associates with ubiquitin in the nucleus and PDLIM2 after interferon treatment

**Fig 4:**
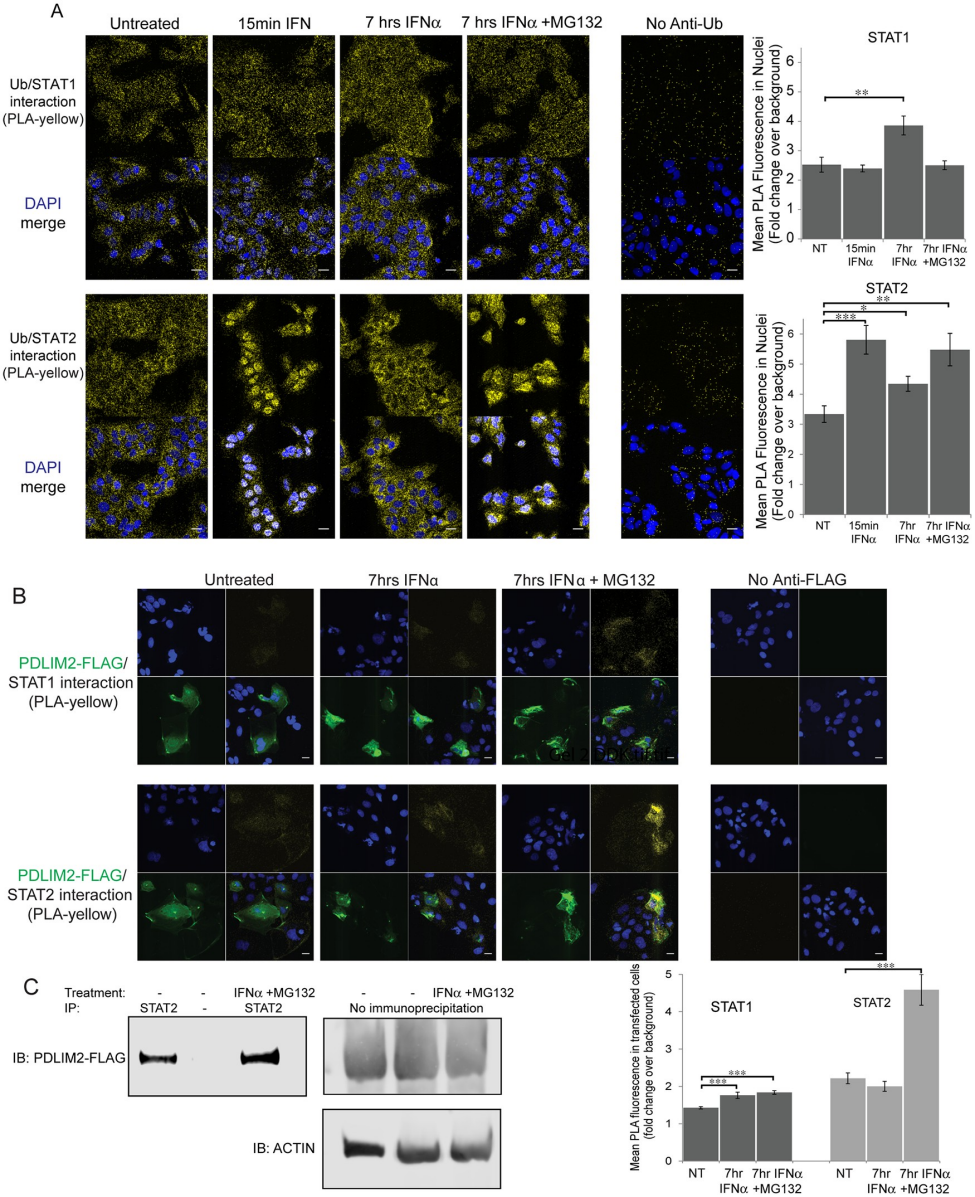
Association of STAT2 but not STAT1 with ubiquitin and PDLIM2 after IFNα treatment. Huh7.5 cells were treated with IFNα2 or both IFNα2 and the proteasome inhibitor MG132 for the indicated times, then fixed. A) Proximity ligation assays (PLA) were performed using STAT1 or STAT2 and polyubiquitin specific antibodies (FK2). Interaction between STAT and ubiquitin in a PLA was visualized in the Cy3 channel (yellow). Nuclei were stained with Hoescht (blue). Quantification of mean fluorescence in cell nuclei was performed on 15 fields per sample, with an average of 42 nuclei per field. B) Huh7.5 cells were first transfected with a pCMV6 vector expressing a FLAG-PDLIM2 fusion protein 2 days prior to treatment with IFNα2 or both IFNα2 and MG132 for 7h, then fixed. Proximity ligation assays (PLA) were performed using STAT1 or STAT2 and FLAG specific antibodies (yellow). Secondary goat anti-mouse Alexa Fluor 488 was used to detect transfected cells expressing FLAG-PDLIM2 (green). Nuclei were stained using Hoescht (blue). Background fluorescence was monitored in a PLA assay lacking either the FLAG or ubiquitin specific antibody (rightmost panels). The mean of yellow PLA fluorescence was measured over an average of 48 transfected cells for each sample. Scale bars are 20 μm. Error bars indicate SEM. Unpaired t-tests were used to determine significance, * denotes p<0.05, ** denotes p<0.01, and *** denotes p<0.001. C) Huh 7.5 cells were co-transfected with plasmids encoding STAT2 and a FLAG tagged PDLIM2. Cells were then untreated or treated with IFNα and MG132 for 7h. Lysates were immuno-precipitated using anti-STAT2 antibodies, separated by SDS-PAGE, and detected using anti-FLAG antibodies. No flag signal was detected when STAT2 antibodies were omitted. Levels of FLAG-PDLIM2 in total lysates are shown.

Since STAT2 was degraded predominantly in the nucleus and PDLIM2 is a ubiquitin E3 ligase, known to degrade NF-κB p65 in the nucleus, we wanted to assess whether STAT2 associates with ubiquitin, where this association occurs and if PDLIM2 is involved in this reaction. We used proximity ligation assays (PLA) to localize the interaction between STAT proteins and ubiquitin. The PLA fluorescent signal is generated if the 2 proteins that the primary antibodies recognize (in this case either STAT1 or STAT2, and ubiquitin) are within 40 nm [69]. We treated Huh7.5 cells with IFN-α for 15 min or 7h, and then examined whether either STAT1 or STAT2 were associated with ubiquitin using PLA (Fig 4A). To assess background, we omitted each primary antibody independently, and the highest background was found when the ubiquitin antibody was omitted. Therefore, the overall fluorescence in the Cy3 channel in a control omitting anti-ubiquitin antibodies (FK2) from the assay was used as background. The Cy3 fluorescent signal produced by PLA in untreated cells was approximately 3 fold higher than that in cells where the primary antibody was omitted. Ubiquitin and STAT1 were associated throughout the cell, and this association did not change location upon IFNα or MG132 treatment. Quantification indicated that there was a small but significant increase in the association between ubiquitin and STAT1 in the nucleus after 7h of IFNα treatment, likely due to an overall increase in the amount of STAT1 caused by IFNα. However, the STAT1/ubiquitin interaction (PLA positive signal) did not reflect the nuclear re-localization of STAT1 either after 15 min of treatment with IFNα, or when MG132 was included during IFNα treatment. In contrast, the interaction between STAT2 and ubiquitin was much more dynamic and reflective of STAT2 localization. Immediately after IFNα treatment, STAT2-ubiquitin association was strikingly nuclear, and by 7h of treatment STAT2-ubiquitin was noticeably absent from the nuclei and increased in the cytoplasm. Consistent with proteasomal degradation of poly-ubiquitinated proteins, inhibition of the proteasome by MG132 resulted in continued STAT2/Ub association in the nucleus. The pattern of STAT2/ubiquitin interaction reflected the pattern of nuclear re-localization of STAT2 seen in response to IFNα treatment. We also performed STAT2 immunoprecipitation to determine if ubiquitin associates with STAT2 when Huh7.5 cells are treated with IFNα in the presence and absence of MG132 for 2h (S6A Fig). The levels of STAT2 increased upon IFNα treatment and STAT2 was not precipitated if anti-STAT2 antibodies were omitted. Immunoblotting of STAT2 immunoprecipitate with anti-ubiquitin antibodies revealed that the levels of polyubiquitined STAT2 increased after treatment with both IFNα and MG132. Polyubiquitin was not precipitated if anti-STAT2 antibodies were omitted. To examine whether HCV infection increases association between STAT2 and ubiquitin within the nuclear matrix, similar to p65, uninfected and HCV infected cells were left untreated, or treated with IFNα for 15 min, then treated and fixed as in Tanaka *et al*. [33] prior to performing a PLA using anti-STAT2 and anti-ubiquitin antibodies (S6B Fig). There was greater association between STAT2 and ubiquitin in the nucleus of HCV infected cells both when untreated or treated with IFNα. Taken together these results are consistent with ubiquitin-dependent STAT2 degradation primarily in the nucleus induced by IFNα treatment.

We next examined whether STAT1 or STAT2 interacted with PDLIM2 after IFNα treatment. We expressed FLAG-tagged PDLIM2 in Huh7.5 cells, treated the cells with IFNα, and examined the interaction of either STAT1 or STAT2 with the tagged PDLIM2 by PLA (Fig 4B). If the anti-FLAG antibody was omitted from the PLA assay there was little background fluorescent signal. Transfected cells (green) expressing tagged PDLIM were identified using anti-FLAG antibodies after the PLA reaction (yellow). There was minimal interaction between STAT1 and tagged PDLIM2 detected by the proximity ligation assay. Again, minimal interaction could be detected between tagged PDLIM2 and STAT2 in untreated cells or in cells treated with IFNα for 7h. However, inhibition of the proteasome with MG132 increased the interaction between STAT2 and PDLIM2 significantly, indicating that inhibition of degradation of STAT2 increased its association with PDLIM2 (Fig 4B). Quantification of PLA indicated that interactions between STAT2 and PDLIM2 increased greater than 2 fold upon interferon treatment in the presence of MG132 whereas the interactions between STAT1 and PDLIM2, while significant, were much lower. These results indicate that interaction between PDLIM2 and STAT2 are increased by IFNα treatment. To further assess the interaction between STAT2 and PDLIM2, STAT2 and PDLIM2-FLAG were expressed in Huh7.5 cells followed by STAT2 immuno-precipitation, and western blotting with anti-FLAG antibodies (Fig 4C). Interactions were detected between STAT2 and PDLIM2-FLAG even in untreated cells, however, treatment with IFNα and MG132 to prevent degradation resulted in increased immuno-precipitation of PDLIM2-FLAG by STAT2 antibodies. Omission of STAT2 antibodies resulted in no PDLIM2-FLAG signal. Taken together these results indicate that IFNα increases the interaction between ubiquitin and STAT2 and also between PDLIM2 and STAT2; however, it does not change STAT1 interactions to the same extent. We postulate that the increased interactions between STAT2 and PDLIM2 lead to ubiquitination of STAT2 and its degradation.

### *PDLIM2* knockout leads to increased levels of STAT2, decreased ubiquitination of STAT2 and increased nuclear retention during IFNα treatment

**Fig 5:**
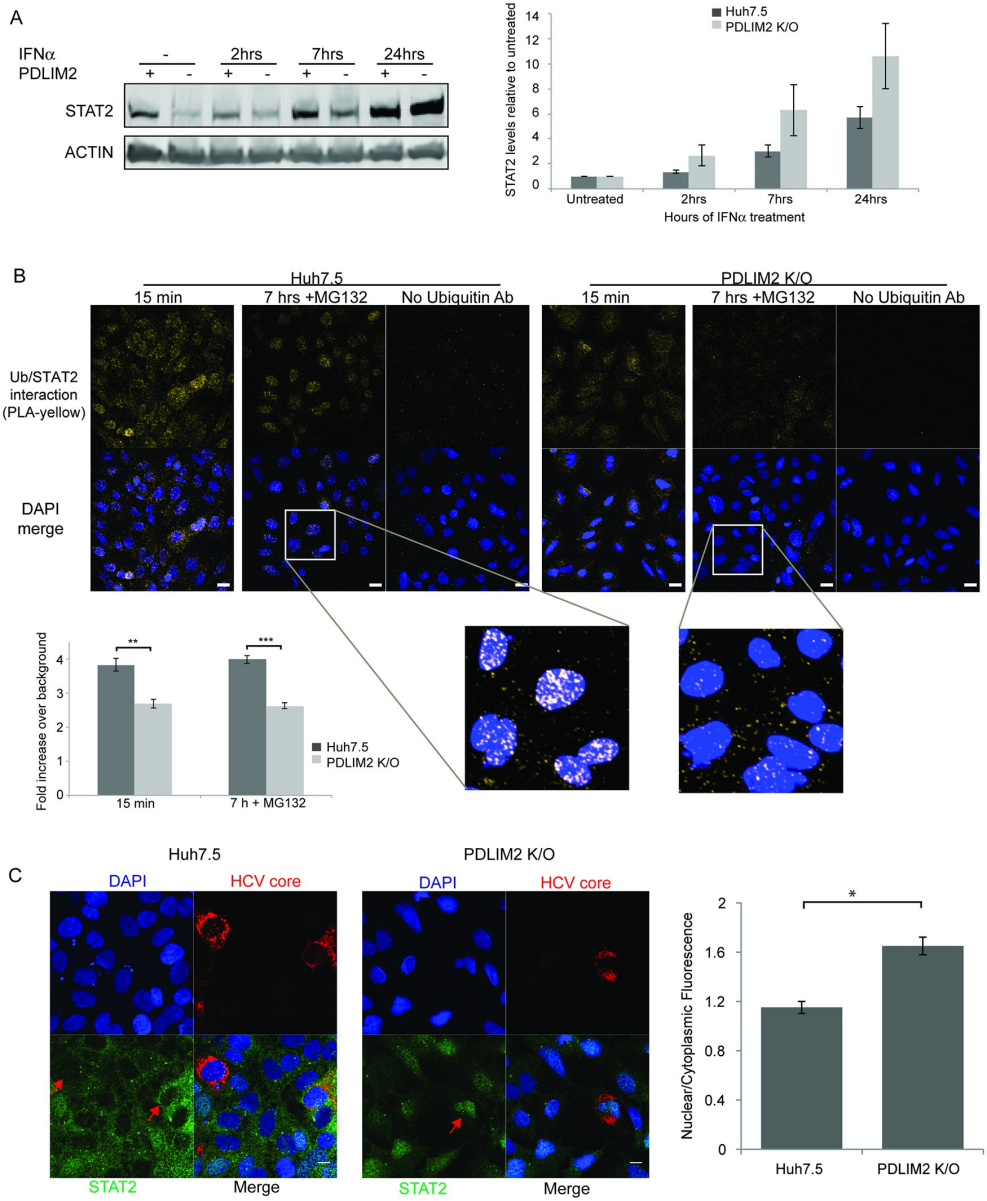
Knockout of *PDLIM2* results in increased STAT2 upon interferon treatment, decreased association with ubiquitin and increased nuclear retention. A) Western blot of STAT2 levels in Huh7.5 and PDLIM2 K/O cells treated with IFNα2 for various times. Overall STAT2 levels examined by Western blot. Quantitation reflects 3 separate experiments. In B) cells were treated with IFNα for either 15 min, or 7h in the presence of cycloheximide and MG132 to prevent new protein synthesis and degradation. They were then treated as in Tanaka *et al*. [33] to reveal interactions in the nuclear matrix, and then interaction between STAT2 and ubiquitin examined by proximity ligation assay. Interaction between STAT2 and ubiquitin in a PLA was visualized in the Cy3 channel (yellow). Nuclei were stained with DAPI (blue). Scale bars are 20 μm. The magnified images were treated identically. Quantification was carried out on a minimum of 214 cells in each condition. Error bars indicate SEM. In C) Huh7.5 and PDLIM2 K/O cells were infected with HCV for 4 days and treated with IFNα for 7h in the presence of cycloheximide to prevent new protein synthesis but without MG132, then fixed and stained for HCV core protein (red) and STAT2 (green). Nuclei were stained using DAPI (blue). Red arrows indicate the nuclei of HCV infected cells. Scale bars are 10 μm. Quantification of the levels of STAT2 in the cytoplasm and the nuclei of 25 of each infected and uninfected cells was done using Velocity software. The ratio of nuclear/cytoplasmic STAT2 staining in Huh7.5 cells and PDLIM K/O cells is shown in the bar graph. Error bars indicate SEM. Unpaired t-tests were used to determine significance, * denotes p<0.05, ** denotes p<0.01, and *** denotes p<0.001.

To determine whether PDLIM2 is a regulator of STAT2 we made a knockout of *PDLIM2* in Huh7.5 cells using the CRISPR/CAS9 system. The knockout is an 800 bp deletion, starting in the first exon of *PDLIM2*, which changes the reading frame of all *PDLIM2* transcripts. Any putative translation products would be truncated and lack the LIM domain needed for ubiquitin ligase activity (S7 Fig). The absence of PDLIM2 expression was confirmed by western blot (S7 Fig). The effects of knocking out PDLIM2 expression are expected to be pleomorphic, as a number of targets for its ubiquitin ligase activity have been proposed [28, 32–35, 58]. Here we have focused on its involvement in the degradation of STAT2 protein in the nucleus of HCV infected cells (Fig 5). We treated either Huh7.5 cells or PDLIM K/O cells with IFNα and examined the levels of STAT2. In untreated cells, STAT2 levels were lower in Huh7.5 cells than in PDLIM K/O cells. However, treatment with IFNα led to greater increases of STAT2 in PDLIM K/O cells than in Huh7.5 cells, ultimately resulting in more STAT2 overall after 24h (Fig 5A). To determine whether PDLIM2 was responsible for STAT2/ubiquitin association, we examined IFNα dependent STAT2/ubiquitin association in the nuclear matrix of Huh7.5 and PDLIM K/O cells using PLA (Fig 5B). Huh7.5 or PDLIM K/O cells were treated with IFNα for either 15 min or 7h to stimulate STAT2 nuclear translocation and degradation. MG132 was included during the 7h incubation to prevent degradation. Ubiquitin was associated with STAT2 in the nuclear matrix of Huh7.5 cells after both 15 min and 7h, whereas the STAT2/ubiquitin association was significantly less in PDLIM K/O cells. We also examined whether *PDLIM2* knockout resulted in nuclear retention of STAT2, similar to what we saw when cells were treated with IFNα while inhibiting the proteasome with MG132 in Huh7.5 cells (Fig 2B). We infected both parental Huh7.5 and PDLIM2 K/O cells with HCV and then treated with IFNα for 7h to stimulate STAT2 nuclear translocation (Fig 5C). In wild type Huh7.5 cells little STAT2 was seen in the nuclei of infected or uninfected cells as we saw in Fig 1B. However, in cells lacking PDLIM2, STAT2 staining was predominantly nuclear in both infected and uninfected cells. This is consistent with what we saw when we inhibited STAT2 degradation during IFNα treatment using MG132 (Fig 2B); however, no MG132 was used. Quantification revealed increased STAT2 in the nucleus of PDLIM K/O cells. Taken together these results indicate that *PDLIM2* knockout results in decreased ubiquitination of STAT2, an increase in STAT2 levels following IFNα treatment and nuclear retention of STAT2 during IFNα treatment. This indicates that in addition to its previously elucidated functions PDLIM2 directs the ubiquitination and degradation of STAT2.

### *PDLIM2* knockout increases IFNα response and decreases viral infection

**Fig 6:**
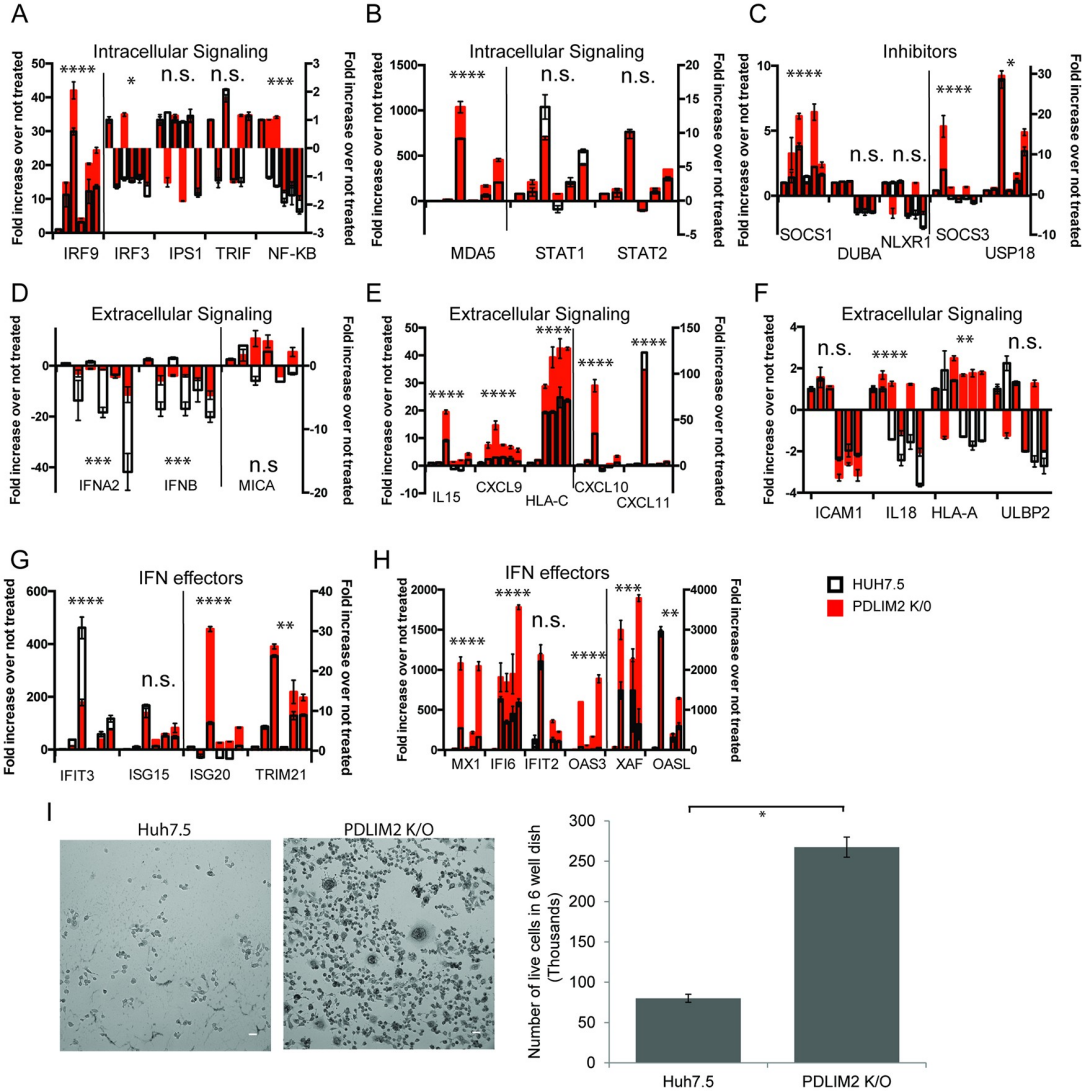
Interferon response in Huh7.5 and PDLIM2 K/O cells. A-H) The expression of 35 ISGs in either Huh7.5 (black outlined bars) or PDLIM knockout cells (red bars) was examined after IFNα2 treatment using a custom TaqMan OpenArray. Six time points are displayed. To examine the initial IFN response, cells were untreated, or treated with IFNα2 for 2 h or 7 h; to examine the subsequent refractory interferon response cells were treated with IFNα2 12 h followed by 12 h rest, then retreated with IFNα2 for 2 h or 7h. A-B) Genes involved in initiation of the innate interferon response, C) inhibitors of the innate immune response. D-F) genes involved in extracellular signaling during the innate interferon response, G-H) effector genes involved in the innate interferon response. HPRT was used to normalize mRNA levels and the average of the duplicate untreated sample was used to determine the fold increase after interferon treatment. Error bars indicate SEM. A 2 way anova analysis was used to determine significance of both IFN addition and the differences between the response seen in Huh7.5 cells and that in PDLIM K/O cells, * denotes p<0.05, ** denotes p<0.01, *** denotes p<0.001, and **** denotes p<0.0001. I) Huh7.5 cells and PDLIM2 K/O cells were infected with VSV for 24 h and then visualized by phase contrast microscopy, or trypsinized, stained with trypan blue and counted. Experiments were performed in duplicate. Scale bars are 40 μm. Error bars indicate standard deviation.

Knockout of PDLIM2 is predicted to have a number of effects because it has many targets that are involved in stimulating the interferon response. We therefore compared the interferon response in the knockout cells to that in Huh7.5 cells using quantitative RT-PCR for a set of ISGs that are induced by HCV infection. For each ISG we examined the response to IFNα at 6 times: untreated, the initial response at 2h and 7h post treatment, then the “so called” refractory interferon response [70, 71]. To induce the refractory response, cells were treated with IFNα for 12h followed by 12h without treatment, and then re-inducing with IFNα for 2h and 7h. These responses are shown as the clusters of six bars for each ISG in Fig 6. We displayed ISGs in groups: those involved in initial intracellular signaling by the innate immune response (Fig 6A–6B), inhibitors (Fig 6C) of the innate immune response, extracellular signals (Fig 6D–6F), and effectors (Fig 6G–6H) of the innate immune response. In general, there was a greater induction of ISG mRNA in the PDLIM2 knockout cells (red bars) than in Huh7.5 cells (white bars), with the exception of IFIT3. There was relatively little induction of, or difference between, Huh7.5 and PDLIM2 K/O cells seen in expression of genes involved in initiation of the innate response or inhibitors (Fig 6A–6C), and some changes were observed in ISGs involved in extracellular signaling (Fig 6D–6F). Most notably, the down regulation of *IFNA2* and *IFNB* mRNA that occurs when exogenous IFNα is added was markedly less in PDLIM2 K/O cells (Fig 6D). The greatest differences between Huh7.5 cells and PDLIM2 K/O cells were in interferon effector genes (Fig 6G and 6H). In some specific genes such as *XAF1*, *OAS3*, *IFI6*, *MX1*, and *HLA-A* the refractoriness of IFNα signaling was abolished in the PDLIM2 K/O cells; however, in most cases the refractory pattern of expression where the expression of a given ISG was lower after the second IFNα stimulation remained approximately the same. When PDLIM2 K/O cells were first transfected with a PDLIM2 overexpression plasmid then treated with IFNα for 7h, the ISG expression was decreased when compared with PDLIM2 K/O cells transfected with the equivalent vector (S7E Fig). These results indicate that overall the response to IFNα is greater in PDLIM2 K/O cells than in parental Huh7.5 cells.

To evaluate whether increased ISG expression in PDLIM2 K/O cells led to a greater antiviral effect, we examined the susceptibility of these cells to vesicular stomatitis virus (VSV) infection because this virus is extremely sensitive to inhibition by the innate immune response [72]. Productive VSV infection leads to cell lysis. Both Huh7.5 cells and PDLIM2 K/O cells were infected with VSV and the extent of cell lysis 24h after infection was used as a measure of productive infection. PDLIM2 K/O cells were significantly more resistant to VSV infection than the parental Huh7.5 cells (Fig 6I). This suggests that the generally increased interferon response detected in our survey of ISGs in PDLIM2 K/O cells is significant enough to inhibit VSV infection.

**Fig 7:**
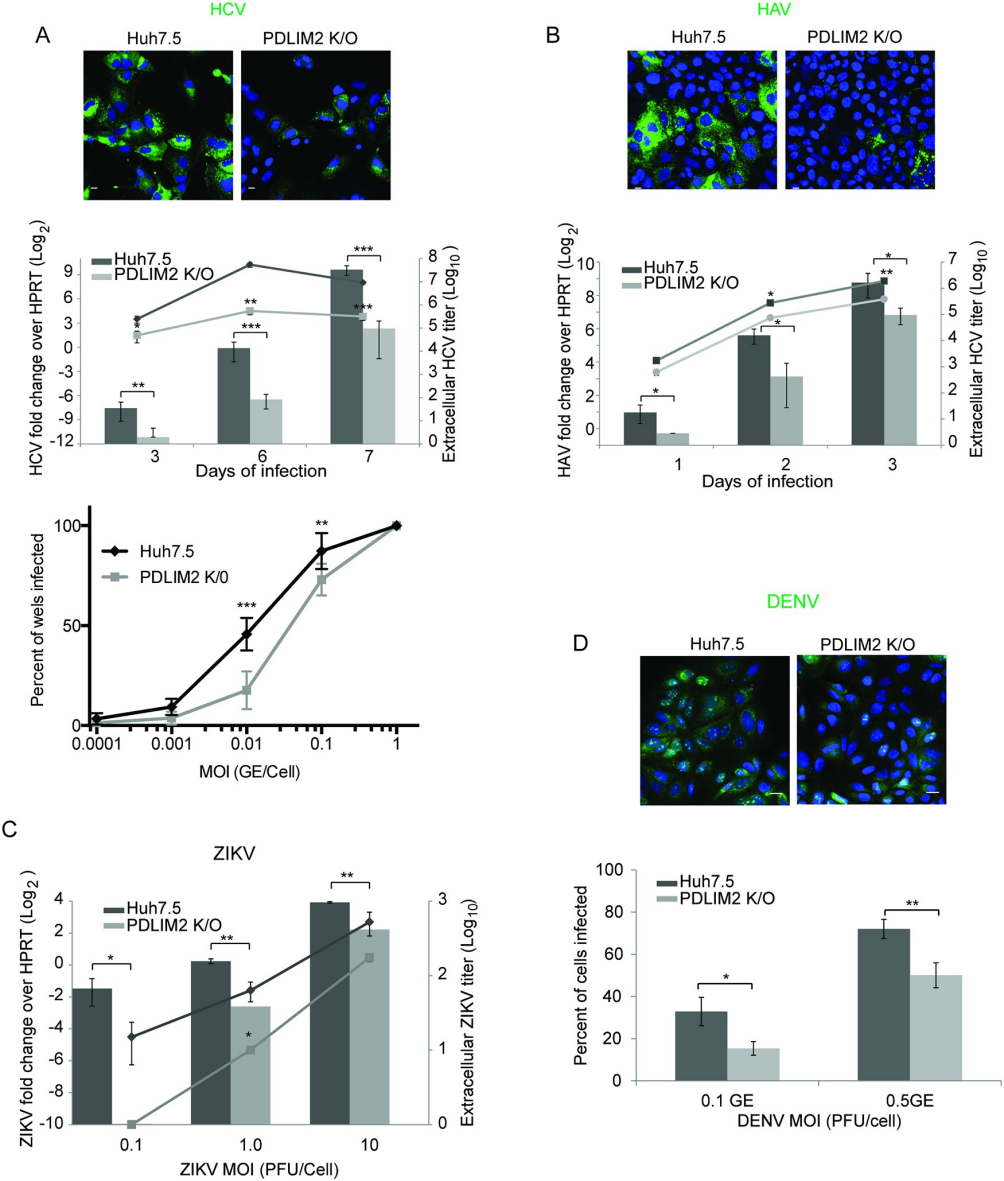
Huh7.5 cells lacking PDLIM2 are more resistant to viral infection than parental Huh7.5 cells. A) HCV: Top Panel: Confocal microscopy of Huh7.5 or PDLIM2 knockout cells infected with 0.1GE/cell of HCV. After 7 days the cells were fixed and HCV core was visualized (green). Nuclei were stained with Hoechst (blue). Scale bars are 10 μm. Middle Panel: Cells were first infected with 1GE/cell of HCV and then qRT-PCR for HCV RNA from cells (bar graph) or supernatants (line graph) was performed after 3, 6, and 7 days of infection. Intracellular HCV RNA levels were normalized to HPRT mRNA levels. Lower Panel: Cells were infected with 8 replicates of the indicated MOI in a 96 well plate. After 4 days cells were stained for the presence of HCV NS5a protein and the percentage of wells containing infected cells was determined. B) Huh7.5 cells and PDLIM2 K/O cells were infected with HAV for 4 days. Cells were fixed and HAV capsid was visualized (Top panel, green) or intracellular HAV RNA quantified by qRT-PCR normalized to HPRT mRNA (Lower panel, bar graph) or extracellular HAV quantified by qRT-PCR (Lower panel, line graph). Experiments were done twice. C) Huh7.5 and PDLIM2 K/O cells were infected with ZIKV at the indicated MOI (pfu/cell) for 24h. Intracellular ZIKV RNA was quantified by qRT-PCR and normalized to HPRT mRNA (bar graph). Extracellular ZIKV was quantified by plaque assay (line graph). Experiments were done 3 times. Error bars indicate SEM. D) Huh7.5 cells or their PDLIM2 K/O derivatives were infected with the indicated MOI of DENV for 36h, fixed, and DENV capsid protein was visualized (Upper panels, green). The percent of DENV infected cells is shown in the bar graph (lower panel). A minimum of 15 fields were counted with an average of 44 cells/field. A representative field at an MOI of 0.1 is shown in the upper panels.

HCV induces a significant interferon response during infection (S1 Table) that it counters by multiple mechanisms. The stronger innate immune response in PDLIM2 K/O cells that we demonstrated above would be predicted to make these cells less susceptible to HCV infection. We therefore investigated the ability of HCV to infect PDLIM2 K/O and Huh7.5 cells (Fig 7A). When infected with identical amounts of virus PDLIM2 K/O cells expressed less HCV core protein and in fewer cells than did Huh7.5 cells when examined by immunofluorescence microscopy (Fig 7A, upper panel). They also had less intracellular HCV RNA and secreted approximately 30 fold less HCV when infected with 1 genome/cell in time course experiments (Fig 7A, middle panel). In a limiting dilution assay (Fig 7A, lower panel), the TCID 50 was lower when determined using Huh7.5 cells than in PDLIM2 K/O cells. Stated differently, it took more virus to infect a similar number of PDLIM2 K/O cells than Huh7.5 cells. Although HCV and HAV are both hepatotropic, HAV infection induces a more limited type I interferon response in host cells than does HCV infection [73, 74]. We therefore examined whether PDLIM2 K/O cells are more resistant to HAV infection (Fig 7B) than parental Huh7.5 cells. Infection with equal amounts of HAV resulted in less HAV core expression, less HAV RNA in cells, and less HAV in the supernatant, indicating that HAV can be controlled by an increased interferon response as expected (Fig 7B). However, it should be noted that the difference in HAV levels between PDLIM2 K/O cells and Huh7.5 cells was smaller than the difference in HCV levels produced in the same cells.

Since we have shown that infection of Huh7.5 cells by ZIKV or DENV increased expression of PDLIM2 (Fig 3) and both viruses are known to degrade STAT2 [67, 68, 75], we examined whether PDLIM2 K/O cells were more resistant to ZIKV or DENV infections. We infected PDLIM2 K/O and parental Huh7.5 cells with several different MOIs of ZIKV. We found that PDLIM2 K/O cells had lower levels of intracellular Zika virus RNA and secreted less Zika virus into culture supernatants than did Huh7.5 cells particularly at low MOI (Fig 7C). In a similar way, we examined whether the PDLIM2 K/O cells were more resistant to DENV infection by infecting both Huh7.5 and PDLIM2 K/O cells with two different MOI. After 36h of infection we stained the cells using DENV capsid specific antibodies and quantified the number of infected cells (Fig 7D). We found that fewer PDLIM2 K/O cells were infected by an equivalent MOI, indicating that cells lacking PDLIM2 are more resistant to both ZIKV and DENV infections. Taken together these results indicate that PDLIM2 is a negative regulator of the interferon response and may be a common target used by a number of flaviviruses to combat the host antiviral response.

## Discussion

The host antiviral response is a complex and highly regulated process, which must be rapid to create a profoundly antiviral state within the infected cell and in surrounding cells under threat of infection. Induction of multiple cytokines during infection is key to resistance to infection. Cross talk between the type I interferon and NF-κB pathways are required for resistance to lethal ectormelia virus infections, and improves response to HCV and hepatitis E virus infection [10, 76]. Consistently, a recent report shows that STAT and NF-κB pathways cooperate by concerted recruitment of the mediator complex to interferon responsive promoters [12]. This coordinated host antiviral response must also be capable of rapid shut down to avoid the detrimental effects of the antiviral state on normal cell function. Prolonged activation of pro-inflammatory pathways is associated with inflammatory and autoimmune diseases [13] as well as the development and proliferation of multiple forms of cancer [77–79]. Inhibition of the NF-κB and STAT pathways can suppress the growth of a number of tumors [80–83]. Given their importance in the innate antiviral response and the connection between HCV and hepatocellular carcinoma, we examined the expression of several key mediators of the host antiviral response, STAT1, STAT2, and NF-κB. We found that all 3 were lower in HCV infected hepatocytes compared with surrounding bystander cells, both *in vivo* in chimeric mouse livers and in Huh7.5 cells, indicating that the interferon response seen during HCV infection is due to uninfected or newly infected hepatocytes [42]. This is consistent with an inverse correlation between HCV RNA and an ISG, IFITM3, found in individual liver cells [54]. We investigated whether lower levels of STAT1, STAT2, and NF-κB in HCV infected cells were due to degradation.

Previous work using mouse cells had shown that PDLIM2/Mystique/SLIM could direct the degradation of NF-κB, STAT1, and STAT4, and that overexpression of PDLIM2 in human cell lines targeted NF-κB p65, STAT1, and STAT4 for ubiquitination and degradation [33, 34, 84]. We found that addition of the proteasome inhibitor MG132 during either IFNα or LPS/IL-1β treatment led to activation and nuclear retention of STAT1, STAT2, or NF-κB. Since PDLIM2 was shown to act in the nucleus following NF-κB activation [33], we investigated the effects of viral infection on PDLIM2 expression in the human hepatocyte cell line Huh7.5. We found that HCV, Zika virus, and Dengue virus infections upregulated PDLIM2 in a dose and time dependent manner, but not in an interferon dependent manner. We next focused our investigations on the mechanism of STAT1 and STAT2 down-regulation in HCV infected cells in the context of interferon treatment. We found that following interferon treatment, STAT2 was degraded in the nucleus but there was little degradation of STAT1. Consistent with the targeting of STAT2 for degradation by PDLIM2, we found that interferon treatment led to association of PDLIM2 with STAT2, but not with STAT1, and interferon treatment led to the ubiquitination of STAT2, but not STAT1 in the nucleus. These results indicate that STAT2 is a target for PDLIM2 in Huh7.5 cells while STAT1 is not, and that targeting STAT2 for degradation may be a common theme among Flaviviruses as has been shown for Zika virus and Dengue virus [66–68, 75] and now HCV. Zika virus NS5 protein interacts with STAT2 to stimulate its degradation while DENV NS5 protein bridges the interaction between STAT2 and UBR4 to stimulate its degradation. We, and others, have shown that HCV NS5A interacts with STAT1 [44]. We speculate by virtue of the ISGF3 complex NS5A may also form a complex with STAT2 and therefore PDLIM2. In our system, overexpression of PDLIM2 led to its association with STAT1, which is consistent with previous reports of STAT1 association with PDLIM2 and its degradation [34, 85]. However, the association between STAT1 and PDLIM2 did not respond to IFNα administration nor did we observe degradation of STAT1 in the presence of cycloheximide, which prevents new protein synthesis. We did observe a lack of induction of STAT1 protein during HCV infection possibly due to the interactions with HCV core [45]. Since STAT1, STAT2 and likely PDLIM2 protein levels vary among cell types and during infection, we cannot rule out that PDLIM2 does not direct interferon independent degradation of STAT1 in other situations. Indeed, a number of IFNα inducible E3 ubiquitin ligases that are known to shut down type I interferon signaling have been reported [86–88]. However, a key component of many efficient and quickly inducible/repressible signaling systems is that of default repression [89], and PDLIM2 is uniquely placed to fulfill this role in type I IFN signaling.

Selective degradation of STAT2 has an additional implication for type I and type II interferon signaling. It has been recently shown that STAT2 inhibits type II interferon signaling [90]. We propose that initial shutdown of the type I interferon response is due to recognition of STAT2 by PDLIM2, followed by ubiquitination, and degradation in a nuclear proteasome, while STAT1 is preserved. We observe that STAT1 is not degraded and is retained in the nucleus when STAT2 degradation is inhibited by MG132. Since STAT1 homodimers bind to gamma activation site (GAS) promoter sequences in response to type II IFN (IFN-γ) stimulation, the preservation of STAT1 may prime the cells to respond to type II interferon as the type I response abates. This mechanism is consistent with previous reports of both the induction of a GAS binding factor and GAS promoter driven transcription by IFN-α and IFN-β [91, 92]. Indeed in separate experiments, we observed that induction of a GAS reporter gene by interferon-γ is more pronounced in PDLIM2 K/O cells than in the parental Huh7.5 cells. Preservation of STAT1 and selective depletion of STAT2 by PDLIM2 during IFNα administration is also consistent with the shift in expression from uniquely IFN-α/β induced proteins at early times after IFN-α treatment, to proteins that are also induced by IFN-γ at later time points [93]. Given the complex activation of STAT proteins and modulation of the IFN response by additional cytokines [94, 95], more complex explanations cannot be ruled out.

Viral promotion of PDLIM2 activity could be a mechanism for premature shut down of the innate immune response. The specific mechanisms used by these viruses to regulate PDLIM2 are not known; however, HCV, ZIKV, and DENV all disrupt the cytoskeleton and both HCV and ZIKV have been proposed to alter the epithelial to mesenchymal transition (EMT) [96–100]. It has been proposed that sequestration of PDLIM2 in the cytosol regulates its function in the nucleus, and that PDLIM2 regulates the EMT [32, 101]. It is possible that disruption of the cytoskeleton during infection results in the nuclear localization that we showed in Fig 3B. Such an alteration would be expected to have pleiotropic effects because this E3 ubiquitin ligase’s known targets, NF-κB and STAT2, and perhaps others occupy key positions in antiviral, proliferative, and apoptotic pathways. NF-κB activation predominantly leads to expression of extracellular cytokines while IFN signaling leads to the activation of intracellular factors [102]. We found that knockout of PDLIM2 and stimulation by IFNα led to activation of genes for both extracellular cytokines and receptors such as CXCL9, 10, HLA-A, and HLA-C, as well as intracellular effector ISGs such as MX-1, OAS3 and ISG20. Intracellular signaling molecules were not as affected. Consistent with an increase in the interferon response in PDLIM K/O cells, viral infection, by a variety of viruses including HCV, ZIKV, DENV and VSV, was attenuated. Taken together these results indicate that PDLIM2 is a key regulator of the innate immune response, and may be manipulated by a variety of viruses to promote their replication. The consequences of dysregulated PDLIM2 expression may range from inflammatory conditions, which during HCV infection, leads to fibrosis and cirrhosis, to the development of tumors in the affected cells [103–108].

## Materials and methods

### Ethics statement

Experimental approval for mouse experiments came from the University of Alberta Animal Welfare Committee according to the Canadian Council on Animal Care guidelines. Study approval #00000348. Prior to harvesting chimeric livers, animals were given isoflurane and put into the surgical plane prior to cervical dislocation.

### Transplantation and infection of chimeric SCID/Alb-uPA mice

All mice were housed VAF and frozen human hepatocytes were purchased from Cellz Direct or ThermoFisher Scientific. Mice were transplanted and infected as described previously [40, 48]. Mice were infected with HCV genotype 2a strain JFH-1 virus and had serum titers greater than 1x10^4^ copies/ml.

### Cells and viruses

Huh7.5 cells (Dr. Charles Rice) were cultured in DMEM (Sigma, D5796) with 10% FBS (Sigma, F1051). Tissue culture adapted JFH-1 was used for all HCV infections, HM175/p16 for HAV infections (Stanley Lemon, University of North Carolina), and PLCal-ZIKV for Zika virus infections. ZIKV, VSV, and Dengue virus were provided by Tom Hobman [68]. Cells were infected for 4-6h at 40%-50% (HCV, DENV), 85% (VSV), or 90% (HAV, ZIKV) confluence and then washed 4x with media. For activation of NF-κB or STAT proteins, recombinant Human IL-1β (PeproTech, 200-01B, 10 ng/ml), LPS (Sigma, 10 ug/ml) or IFNα2b (Schering, 02238675, 1000 IU/mL)or IFN-λ3 (R&D Systems, 5259IL-025) were diluted in media immediately before use. Cycloheximide (Sigma, 7698, 50 μM) and MG132 (Sigma, 2211, 10 μM) were added in conjunction as indicated.

### Antibodies

Commercially available antibodies were used to detect FK2 (Enzo Life Sciences, BML-PW8810), DDK tag (Origene, TA50011), HCV NS3 (TORDJI-22, Abcam), HCV core (ThermoFisher Scientific, MA1-080), HAV capsid (Commonwealth Serum Laboratories, K24F2), Dengue capsid (Dr. Tom Hobman, University of Alberta), STAT1 (Cell Signaling Technologies, CST9175), STAT2 (Santa Cruz, sc-476), Y701-phosphoSTAT1 (Cell Signaling Technologies, 9167S), Y690-phosphoSTAT2 (Cell Signaling Technologies, 88410S), PDLIM2 (Abcam, 246868), p65 NF-κB (Santa Cruz, sc-372), Actin (EMD Millipore, MAB1501), PARP-1 (BD Pharmingen, BD556362), Lamin (Zymed, 33–2000), B-tubulin (Abcam, AB6046), Anti-NS5a (gift from Charlie Rice, 9E10). For western blotting, goat anti-mouse IR Dye 800 (Licor, 926–32210) and goat anti-rabbit IR Dye 680 (Licor, 926–32221) were used. For immunofluorescence, Alexa Fluor 546 goat anti-mouse IgG (ThermoFisher Scientific, A11030), Alexa Fluor 488 goat anti-rabbit IgG, (ThermoFisher Scientific, A11008), or Alexa Fluor 647 goat anti-mouse IgG (ThermoFisher Scientific, A21236) were used.

### Primers and probes

Custom primer/probe sets were used for HCV, HAV, and ZIKV (S2 Table). TaqMan assays (ThermoFisher Scientific) were used for PDLIM2 (Hs00917389_m1), and HPRT (Hs99999909_m1). For the interferon response gene panel, a Gene Expression MicroFluidics Card with a custom Human ISG array from Applied Biosystems was used. See S2 Table for genes and assay numbers. Additionally, Primers and probes for *CXCL9*, *CXCL10*, *IFI6*, *IRF9*, *MX1*, *SOCS1*, and *XAF* (S2 Table) were used to assess ISG response using IDT Prime Time quantitative RT-PCR assays when PDLIM2 K/O cells were transfected with PDLIM2.

### Cell fractionation and western blots

Cells were collected using Accutase (Millipore, SCR005) and lysed in Cytoplasmic Extract buffer (CE) (10mM HEPES, 10mM KCl, 0.1mM EDTA, 0.1 mM EGTA pH8, 0.1% Triton X-100, Sigma Protease Inhibitor 11873580001). After 40 minutes gentle rotation at 4°C, cytoplasmic fractions were separated from nuclear fractions by centrifugation at 1000x g for 5 minutes and then collected. Nuclear pellets were washed 4 times in CE buffer, then lysed with Nuclear Extract buffer (20mM HEPES, 25% glycerol, 0.5M NaCl, 1mM EDTA, 1mM EGTA, Sigma Protease Inhibitor). For phosphoSTAT western blots, cells were collected in Eppendorf tubes by scraping on ice in ice cold Radioimmunopercipitation assay buffer (RIPA) (50mM Tris pH 7.4, 150mM Sodium Chloride, 0.5% Sodium Deoxycholate, 1% Nonidet P-40) supplemented with 1mM Sodium-Orthovanadate, 10mM Beta-Glycerophosphate, 50mM Sodium Fluoride, and 1x Protease Inhibitor Cocktail (Sigma Aldrich). Samples were allowed to incubate at -20°C for 15 min, after which they were briefly vortexed and clarified at 14,000g for 10 min at 4°C. Supernatants were collected and 5x Protein Sample Buffer (250mM Tris-Cl pH6.8, 5% SDS, 45% Glycerol, 9% Beta-Mercaptoethanol, 0.01% Bromophenol Blue) was added to result in 1x samples for western blotting. For immunoprecipitation: Huh7.5 cells were lysed as in [33] and then incubated at 22°C with rabbit anti STAT2 antibodies for 5.5 h, then incubated with sheep anti-rabbit IgG Dynabeads (Thermo, 11204D) overnight at 4°C, followed by 8 washes with 50mM Tris-HCL pH 7.5, 150mM NaCl, 0.05% Triton X-100. Western blotting was performed as per standard methods [109]. Signal was detected with an Odyssey Infrared Imaging system (Licor).

### Transfection

Cells were transfected using Lipofectamine 2000 (ThermoFisher Scientific, 11668019) according to manufacturer’s instructions. The GFP-tagged STAT2 construct and the DDK-tagged PDLIM2 construct was purchased from Origene (RG208592, accession number NM_005419.2 and RC210022, accession number NM_021630.4). The PDLIM2 construct corresponds to PDLIM2 transcript variant 2. For comparison of ISG levels after 7h of IFNα treatment PDLIM2 K/O cells were transfected with the DDK tagged PDLIM2 construct or an equivalent construct expressing a GFP-GFP fusion protein 24h prior to IFNα treatment (Viromer Red - Lipocalyx, VR-01LB-00) essentially according to the manufactures protocol except twice the recommended amount of plasmid was used. The control was a CMV expressed double GFP [110].

### PDLIM2 knockout

PDLIM2 K/O cells were generated from Huh7.5 cells. A homozygous deletion between positions 6914 and 7715 of the PDLIM2 gene (RefSeq NG_030435.1) was generated by CRISPR-based gene editing following the protocol of Ran *et al* [111]. Two target sequences within the gene (CCCTGGGGCTTCCGTATCAC and CATCAACGGGGAAAGCGCGG) were each inserted into the vector pSpCas9 (BB)-2A-Puro v2.0 (Addgene, PX459). The two constructs were co-transfected into Huh7.5 cells using GenJet reagent (II) (SL100489-HUH). After 3 days of selection in media containing 2 ug/mL puromycin, live cells were selected by flow cytometry. Serial dilutions of the cells were plated on mitomycin C-inactivated NIH-3TC feeder cells. An initial clone, C4, was subcloned by rounds of serial dilution until a clone was obtained that, by PCR and sequence analysis, showed precise deletion of the sequences between the two predicted CRISPR-CAS9 cleavage sites in the PDLIM2 gene (S4 Fig). The deletion alters the reading frame of all PDLIM2 transcripts such that putative translation products would be truncated near their N-termini; they would have a truncated PDZ domain implicated in protein:protein interactions and lack the LIM domain needed for ubiquitin E3 ligase activity.

### Immunofluorescence

Huh7.5 cells grown on glass coverslips were fixed with 1:1 methanol: acetone at -20°C for a minimum of 30 minutes, then washed with PBS and blocked for 1hr in PBS with 1% bovine serum albumin (Sigma, A3912) and 2.5 mM EDTA. For STAT1/STAT2 IF, cells were fixed with 4% paraformaldehyde for 40 min, permeabilized in 0.1% saponin in PBS for 40 min, blocked 1% BSA/2.5 mM EDTA/0.1% saponin for 1hr, and washed with PBS/0.1% saponin. Cells were incubated with primary and secondary antibodies (see above), diluted in block for 1hr at room temperature. Following secondary antibody application, cells were mounted with DAPI Fluoromount-G (Southern Biotech, 0100–20), or incubated with 1/5000 Hoechst 33342 (ThermoFisher Scientific) in PBS for 5 minutes, then washed and mounted on slides with Vectashield Mounting Medium for Fluorescence (H-1000) and sealed with nail polish. Confocal images were obtained using a Quorum Wave FX-2 spinning disk microscope using 20X/0.85 NA or 40x/1.3NA oil immersion lenses. Quantification was done using ImageJ (NIH) or Velocity 6.2.1 (PerkinElmer). Brightfield photos were obtained using a Zeiss Axiovert 200M microscope with a Fluar 10x/0.5 NA lens.

### Proximity ligation assay

Samples were treated as described above for growing, fixation, blocking, and primary antibody incubation, except for coverslips prepared for nuclear matrices, which were first treated as described by Tanaka *et al*. [33], then treated as above. Following primary antibody incubation, a Duolink proximity ligation assay kit (Sigma, DUO92102) was used according to manufacturer’s instructions except that following the final wash coverslips were immediately mounted onto slides rather than being allowed to dry.

### RNA purification and quantitative real-time PCR

Extracellular HAV and HCV RNA were purified using the Roche High Pure Viral Nucleic Acid kit (11858874001) as per manufacturer’s instructions. cDNA was synthesized with Superscript III (ThermoFisher Scientific, 18080044) and random primers (for HAV, ThermoFisher Scientific, 48190011) or a gene-specific primer for HCV (HCV reverse primer, see above). Intracellular RNA was harvested using QIAzol (Qiagen, 79306) according to manufacturer’s instructions, and cDNA was synthesized using M-MLV (ThermoFisher Scientific, 28025013). Prime Time (IDT) PCR assay mix was used with for qRT-PCR using primers listed in S2 Table.

### ZIKV plaque assay

Extracellular ZIKV was measured by plaque assay on Vero cells (Dr. Tom Hobman, University of Alberta). ZIKV virus was diluted in DMEM to the MOI indicated, and the media applied to Huh7.5 or PDLIM2 K/O cells. After 1h, media was removed, cells washed in DMEM, and fresh DMEM + FBS (10%) applied. Supernatant was harvested after 1d, 2d, or 3d, and applied to Vero cells for 1h, after which media was removed, cells washed, and media containing 1.5% carboxymethylcellulose (Sigma-Aldrich, 21902) added. After 4 days, paraformaldehyde (PFA) was added to the media to a final concentration of 5% PFA for 30 minutes. Following fixation, a 0.1% crystal violet solution was used to stain the plaques.

## Supporting information

S1 Fig Confocal microscopy isotype controls and STAT/HCV staining in HCV infected chimeric human/mouse liver. Confocal microscopy was performed on HCV infected liver sections. A) Liver sections containing human hepatocytes stained using primary mouse IgG_1_ isotype control and nonspecific rabbit IgG antibodies (first panel) or antibodies directed against HCV and rabbit polyclonal antibodies specific for human STAT2 (middle panel). The last panel shows an area of the chimeric liver containing only mouse hepatocytes and stained as in the middle panel. B) Additional fields stained with antibodies specific for either STAT1 or STAT2 and HCV in the same manner showing both infected and uninfected cells within an infected liver. The nuclei were stained with DAPI, and mouse antibodies were visualized using secondary goat anti mouse-HRP and tyramide -TMR substrate (red). Secondary goat anti-rabbit Alexa 488 antibodies (green) were used to visualize the STAT proteins. The scale bars are shown.
(TIF)

S2 Fig Evaluation of NF-κB p65 levels in HCV infected humanized chimeric mouse liver cells by confocal microscopy. Confocal microscopy was performed on either uninfected (A) or HCV infected human hepatocytes (B and C) in chimeric human/mouse liver sections. Sections were stained using mouse monoclonal antibodies directed against HCV NS3 (red) and rabbit polyclonal antibodies specific for human NF-κB p65 (green). The nuclei were stained with DAPI, and mouse antibodies were visualized using secondary goat anti-mouse-HRP and tyramide-TMR substrate. Secondary goat anti-rabbit Alexa Fluor 488 antibodies were used to visualize NF-κB p65. The scale bars are 10μm. Isotype controls are depicted in S1 Fig. Panel A and B were done at the same time with identical laser settings and exposures while panel C was done later.
(TIF)

S3 Fig STAT1 and STAT2 levels in uninfected and HCV infected cells. A) Huh7.5 cells were uninfected or infected with 3 and 10 genome equivalents of HCV for 4 days and the levels of STAT1 and STAT2 were determined by western blot. B-tubulin was used as a loading control. B) Huh7.5 cells left uninfected or were infected with 10 genome equivalents/cell and then treated with cycloheximide to prevent new protein synthesis and then either IFNα to stimulate STAT phosphorylation and nuclear translocation or both IFNα and MG132 to prevent protein degradation for 12h. C) HCV infected cells (3 GE/cell for 4 days) were either left untreated or treated with IFNα for 12h, fixed and stained with antibodies specific for HCV core (green). Nuclei were stained with DAPI. Images shown are 9x9 stitched images, and scale bars are 60 μm. The quantitation shown is based on a minimum of 333 cells for each condition. Error bars are SEM. Unpaired t-tests were used to determine significance. The levels of HCV core in cells infected with 10 GE/cell also don’t change during IFNα treatment.
(TIF)

S4 Fig Evaluation of NF-κB p65 levels in IL1β/LPS treated HCV infected Huh7.5 cells by confocal microscopy. Confocal microscopy was performed on Huh7.5 cells that were uninfected or infected with HCV JFH-1 (3 GE/cell) for 4 days, and either untreated or treated with 10 ng/mL IL1β and 10 μg/mL LPS for the indicated times or IL1β/LPS alongside the proteasome inhibitor MG132, then fixed. Cells were stained using mouse monoclonal antibodies directed against HCV core (red) and rabbit polyclonal antibodies specific for NF-κB p65 (green). Nuclei were stained with Hoescht (blue). HCV core was visualized using secondary goat anti-mouse Alexa Fluor 546. NF-κB was visualized using secondary goat anti-rabbit Alexa Fluor 488 antibodies. The scale bars are 120μm.
(TIF)

S5 Fig Confocal microscopy of cells infected with HCV (A), DENV (B) and ZIKV (C) at a variety of MOI. Huh7.5 cells were infected with differing amounts of HCV, DENV and ZIKV for 4, 2, and 2 days, respectively, followed by fixation, staining with HCV core, or ZIKV capsid or DENV capsid specific antibodies (green), and visualization by fluorescence confocal microscopy. The nuclei are stained with DAPI (blue), and scale bars are 20 μm, except for ZIKV where they are 10 μm.
(TIF)

S6 Fig HCV infection increases association between STAT2 and ubiquitin in the nucleus. A) STAT2 immunoprecipitation. Huh7.5 cells were left untreated or treated with IFNα and MG132 for 2h. Lysates were immuno-precipitated using anti-STAT2 antibodies, separated by SDS-PAGE, and detected using anti-FK2 antibodies. No signal was detected when STAT2 antibodies were omitted. Levels of ubiquitin total lysates are shown. STAT2 immunoblots are also shown. B) Uninfected or HCV infected Huh7.5 cells were treated with IFNα2 for 15 min then treated with CSK and extraction buffer as in Tanaka *et al*. [33], then fixed. Proximity ligation assays (PLA) were performed using STAT2 and polyubiquitin specific antibodies (FK2). Interaction between STAT2 and ubiquitin in a PLA was visualized in the Cy3 channel (yellow). The magnified images of untreated uninfected and untreated HCV infected cells were manipulated identically and are included for clarity. Nuclei were stained with Hoescht (blue). Scale bars are 20 μm. Quantification of mean fluorescence in cell nuclei was performed on a minimum of 246 cells. Error bars indicate SEM. Unpaired t-tests were used to determine significance, *** denotes p<0.001.
(TIF)

S7 Fig PCR and sequencing characterization of PDLIM2 K/O cells. CRISPR/Cas9 was used to direct cleavage at 2 sites, the first in exon 1 and the second in exon 2 of the *PDLIM2* gene as described in Materials and Methods. Blunt end ligation of DNA cleaved at both sites by cellular repair pathways yielded a deletion at the *PDLIM2* locus in some cells. A clonal line of cells containing this deletion was isolated and characterized. A) PCR amplification products using primers from within the deleted region using template DNA from: parental Huh7.5 cells, an initial *PDLIM2* deletion clone with some wild type Huh7.5 contamination (C4), a purified subclone of C4 designated the PDLIM K/O cell line, and a no template control. No trace of the deleted region could be detected in the purified clone. B) Sequence at the deletion breakpoint. T6914 and C7715 of the *PDLIM2* gene (RefSeq NG_030435.1) have been ligated in the PDLIM2 K/O derivative of Huh7.5 with the deletion of the intervening 800 bp on both chromosomes. C) Diagram of *PDLIM2* exons and the position of the deletion between the first and second exons, and the resulting presumed protein sequence. D) Western blot of MA104, Huh7.5, and PDLIM2 K/O cells with antibodies specific to PDLIM2. E) Interferon response in PDLIM K/O cells transfected with either a vector expressing double GFP protein (black), or PDLIM2 (red). PDLIM K/O cells were transfected 24h prior to treatment for an additional 7h with IFNα, lysed in Qiazol, RNA isolated and qRT-PCR was performed using PrimeTime qRTPCR assays (IDT) for 7 ISGs (S2 Table).
(TIF)

S1 Table Interferon response gene expression in Huh7.5 cells infected with HCV. Huh7.5 cells either uninfected or infected with HCV JFH-1 for 2, 4 or 7 days in duplicate wells (A and B) were lysed in Qiazol, RNA isolated, and qRT-PCR performed using a custom TaqMan OpenArray. The results for 41 genes, predominantly ISGs, are shown. The CT values were standardized to HPRT mRNA levels and the average of the duplicate uninfected sample was used to determine the fold increase in ISG gene expression after infection.
(PDF)

S2 Table Genes and ABI assay numbers for the TaqMan custom PCR array and primer sequences for qRT-PCR. The genes and assay numbers from the Gene Expression MicroFluidics Card with a custom Human ISG array from Applied Biosystems are shown.
(PDF)
