# Supplementary figures and images for: HCV and flaviviruses hijack cellular mechanisms for nuclear STAT2 degradation: Up-regulation of PDLIM2 suppresses the innate immune response
Source: PLoS Pathog. 2019 Aug 2;15(8):e1007949. doi: 10.1371/journal.ppat.1007949 (PMC6677295; doi:10.1371/journal.ppat.1007949)

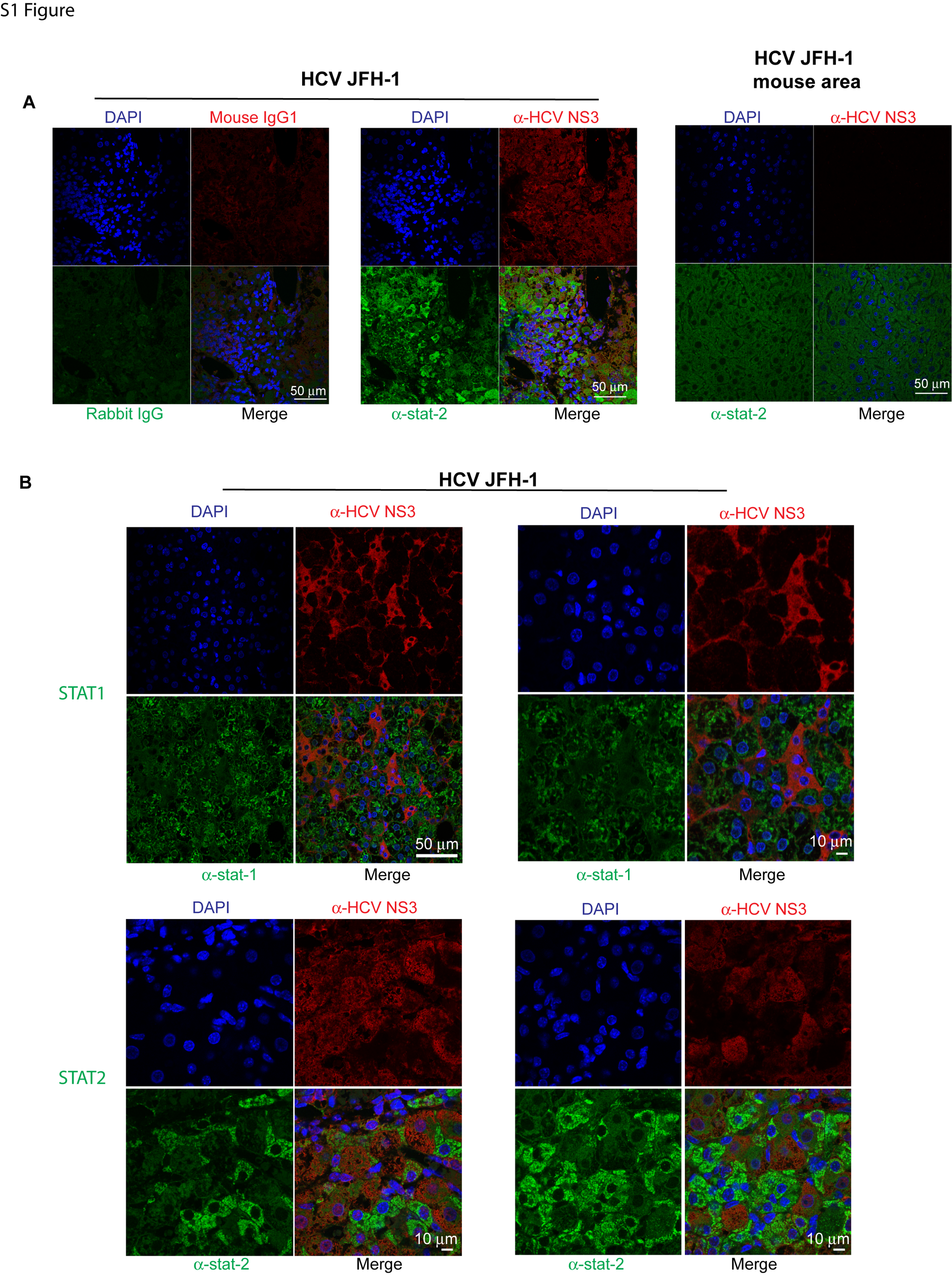

Supplement: S1 Fig — Confocal microscopy was performed on HCV infected liver sections. A) Liver sections containing human hepatocytes stained using primary mouse IgG1 isotype control and nonspecific rabbit IgG antibodies (first panel) or antibodies directed against HCV and rabbit polyclonal antibodies specific for human STAT2 (middle panel). The last panel shows an area of the chimeric liver containing only mouse hepatocytes and stained as in the middle panel. B) Additional fields stained with antibodies specific for either STAT1 or STAT2 and HCV in the same manner showing both infected and uninfected cells within an infected liver. The nuclei were stained with DAPI, and mouse antibodies were visualized using secondary goat anti mouse-HRP and tyramide -TMR substrate (red). Secondary goat anti-rabbit Alexa 488 antibodies (green) were used to visualize the STAT proteins. The scale bars are shown. (TIF) [file ppat.1007949.s001.tif]

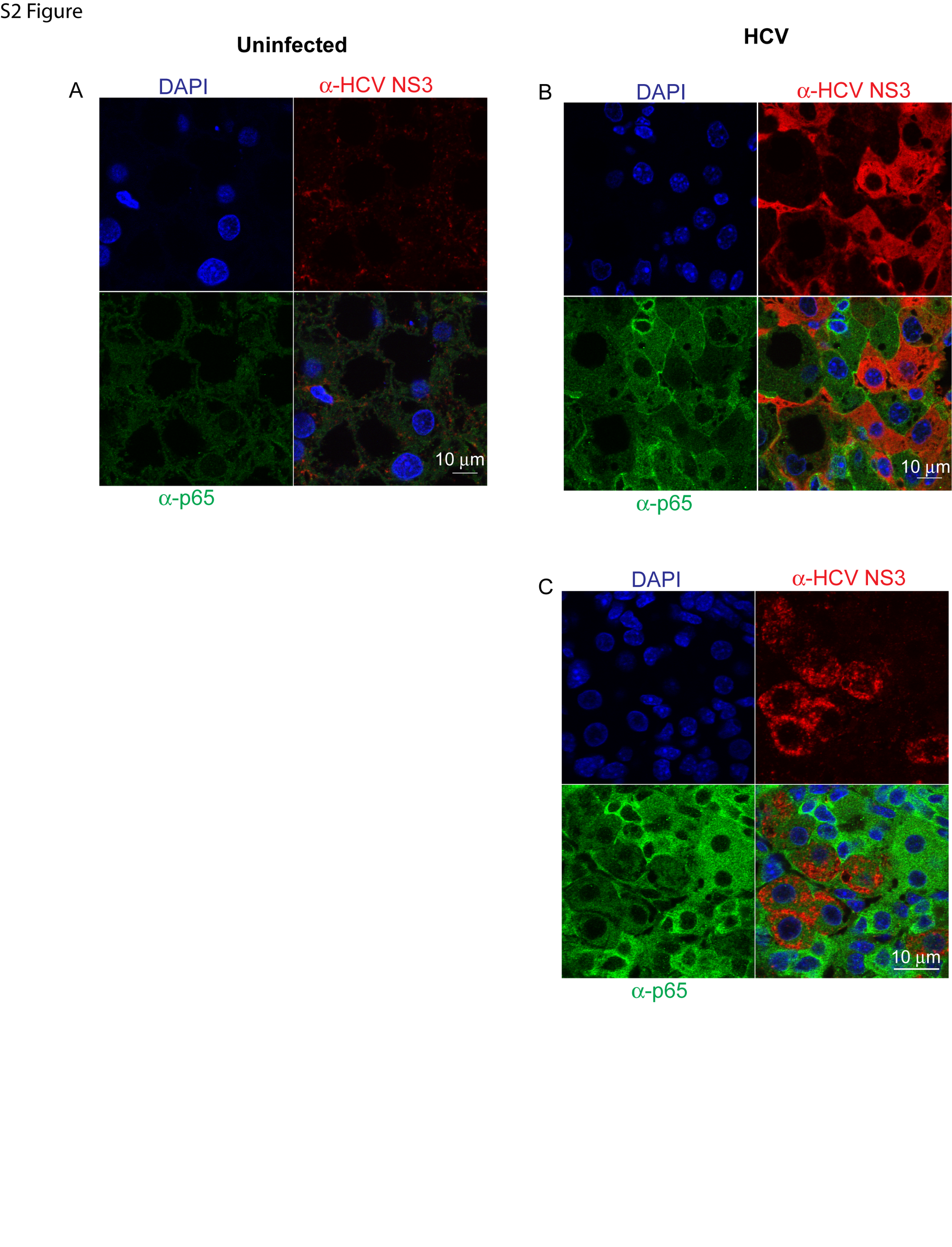

Supplement: S2 Fig — Confocal microscopy was performed on either uninfected (A) or HCV infected human hepatocytes (B and C) in chimeric human/mouse liver sections. Sections were stained using mouse monoclonal antibodies directed against HCV NS3 (red) and rabbit polyclonal antibodies specific for human NF-κB p65 (green). The nuclei were stained with DAPI, and mouse antibodies were visualized using secondary goat anti-mouse-HRP and tyramide-TMR substrate. Secondary goat anti-rabbit Alexa Fluor 488 antibodies were used to visualize NF-κB p65. The scale bars are 10μm. Isotype controls are depicted in S1 Fig. Panel A and B were done at the same time with identical laser settings and exposures while panel C was done later. (TIF) [file ppat.1007949.s002.tif]

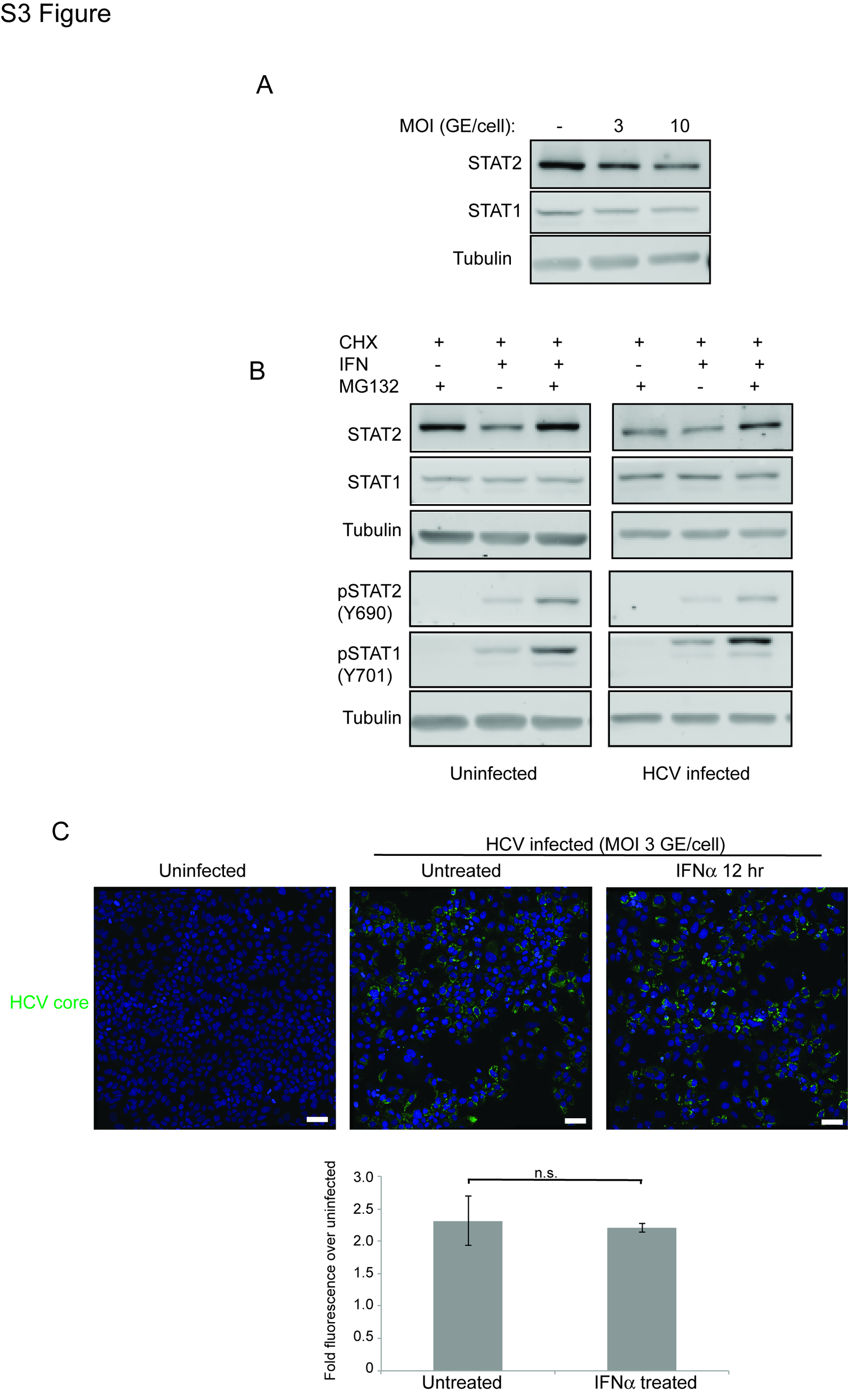

Supplement: S3 Fig — A) Huh7.5 cells were uninfected or infected with 3 and 10 genome equivalents of HCV for 4 days and the levels of STAT1 and STAT2 were determined by western blot. B-tubulin was used as a loading control. B) Huh7.5 cells left uninfected or were infected with 10 genome equivalents/cell and then treated with cycloheximide to prevent new protein synthesis and then either IFNα to stimulate STAT phosphorylation and nuclear translocation or both IFNα and MG132 to prevent protein degradation for 12h. C) HCV infected cells (3 GE/cell for 4 days) were either left untreated or treated with IFNα for 12h, fixed and stained with antibodies specific for HCV core (green). Nuclei were stained with DAPI. Images shown are 9x9 stitched images, and scale bars are 60 μm. The quantitation shown is based on a minimum of 333 cells for each condition. Error bars are SEM. Unpaired t-tests were used to determine significance. The levels of HCV core in cells infected with 10 GE/cell also don’t change during IFNα treatment. (TIF) [file ppat.1007949.s003.tif]

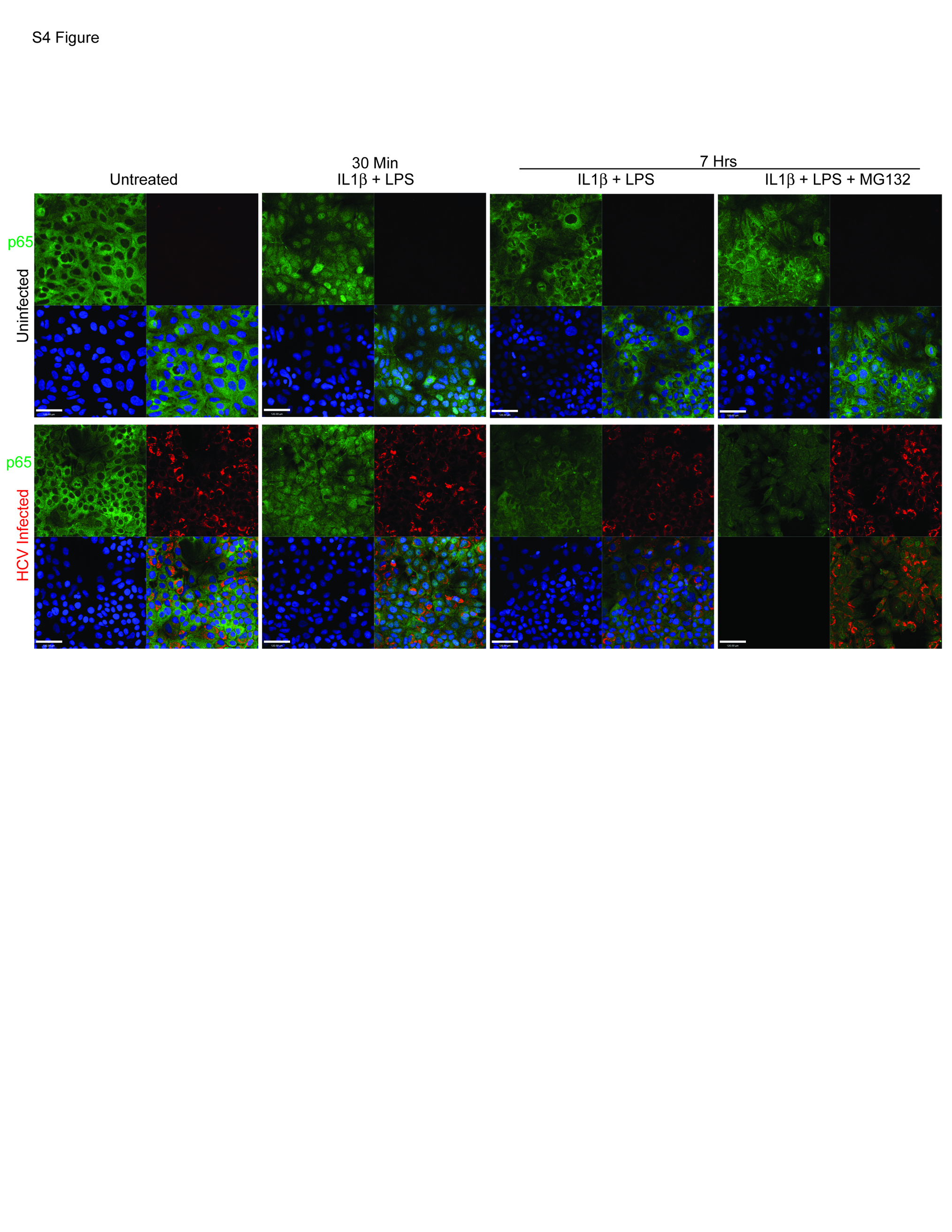

Supplement: S4 Fig — Confocal microscopy was performed on Huh7.5 cells that were uninfected or infected with HCV JFH-1 (3 GE/cell) for 4 days, and either untreated or treated with 10 ng/mL IL1β and 10 μg/mL LPS for the indicated times or IL1β/LPS alongside the proteasome inhibitor MG132, then fixed. Cells were stained using mouse monoclonal antibodies directed against HCV core (red) and rabbit polyclonal antibodies specific for NF-κB p65 (green). Nuclei were stained with Hoescht (blue). HCV core was visualized using secondary goat anti-mouse Alexa Fluor 546. NF-κB was visualized using secondary goat anti-rabbit Alexa Fluor 488 antibodies. The scale bars are 120μm. (TIF) [file ppat.1007949.s004.tif]

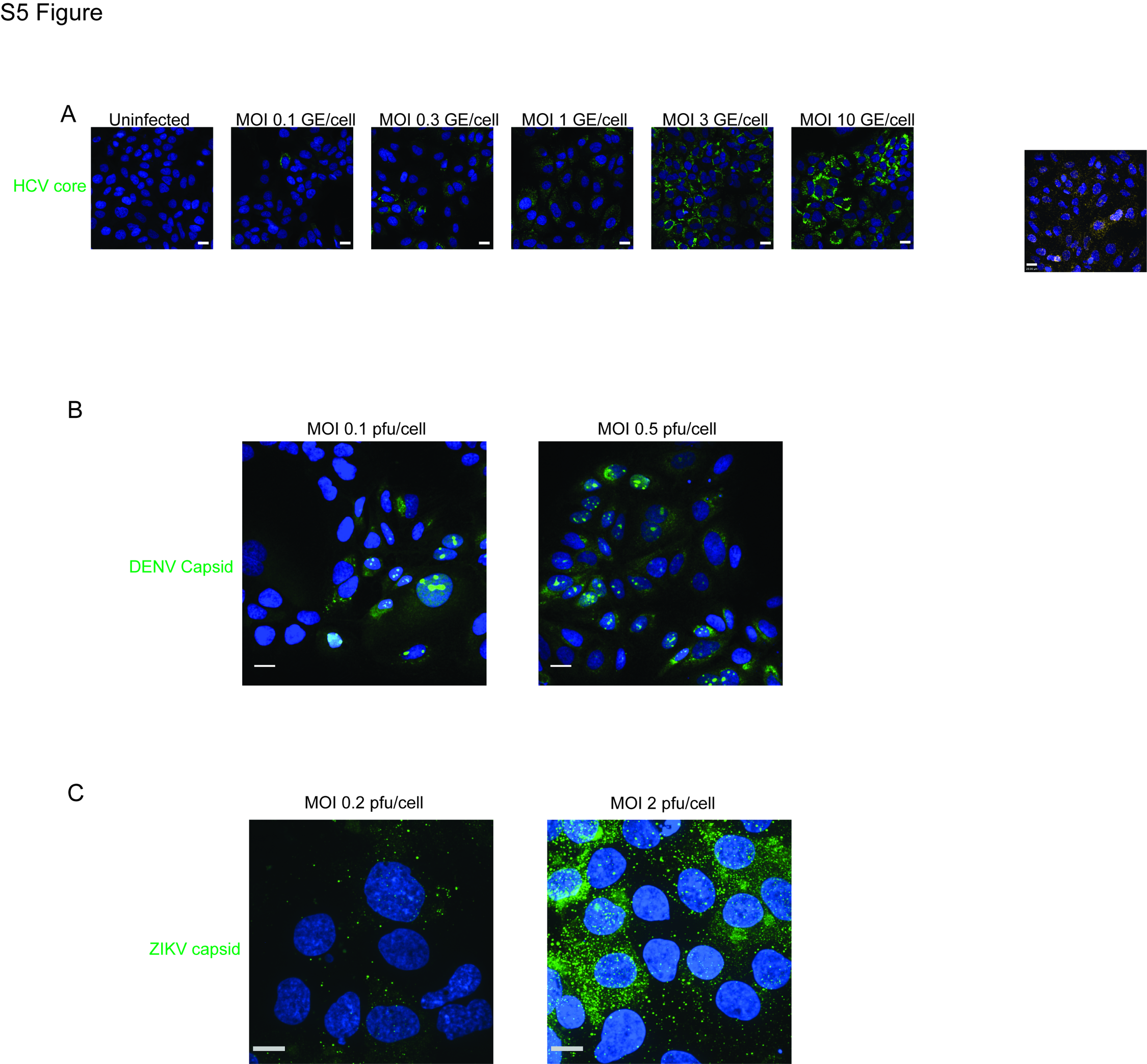

Supplement: S5 Fig — Huh7.5 cells were infected with differing amounts of HCV, DENV and ZIKV for 4, 2, and 2 days, respectively, followed by fixation, staining with HCV core, or ZIKV capsid or DENV capsid specific antibodies (green), and visualization by fluorescence confocal microscopy. The nuclei are stained with DAPI (blue), and scale bars are 20 μm, except for ZIKV where they are 10 μm. (TIF) [file ppat.1007949.s005.tif]

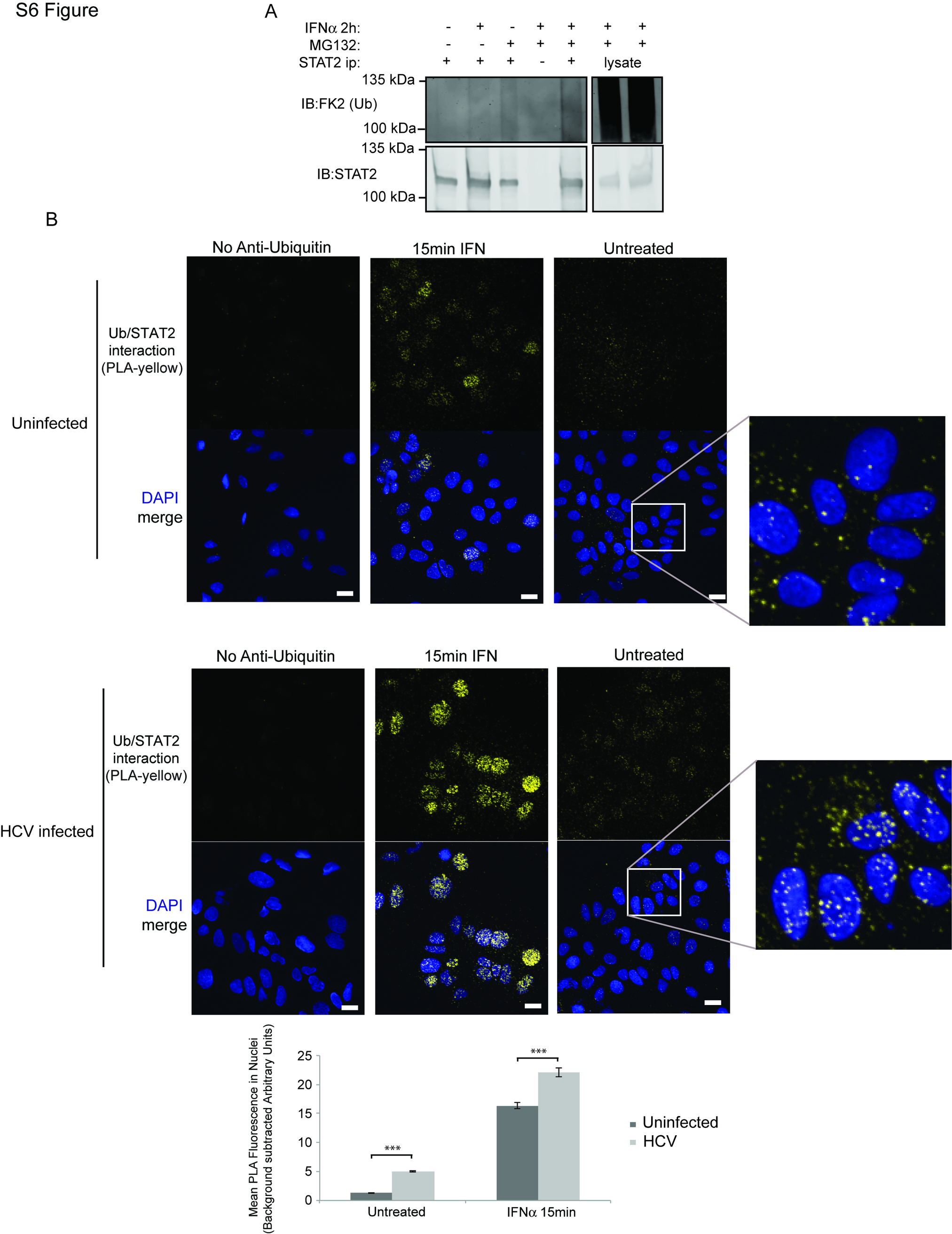

Supplement: S6 Fig — A) STAT2 immunoprecipitation. Huh7.5 cells were left untreated or treated with IFNα and MG132 for 2h. Lysates were immuno-precipitated using anti-STAT2 antibodies, separated by SDS-PAGE, and detected using anti-FK2 antibodies. No signal was detected when STAT2 antibodies were omitted. Levels of ubiquitin total lysates are shown. STAT2 immunoblots are also shown. B) Uninfected or HCV infected Huh7.5 cells were treated with IFNα2 for 15 min then treated with CSK and extraction buffer as in Tanaka et al. [33], then fixed. Proximity ligation assays (PLA) were performed using STAT2 and polyubiquitin specific antibodies (FK2). Interaction between STAT2 and ubiquitin in a PLA was visualized in the Cy3 channel (yellow). The magnified images of untreated uninfected and untreated HCV infected cells were manipulated identically and are included for clarity. Nuclei were stained with Hoescht (blue). Scale bars are 20 μm. Quantification of mean fluorescence in cell nuclei was performed on a minimum of 246 cells. Error bars indicate SEM. Unpaired t-tests were used to determine significance, *** denotes p<0.001. (TIF) [file ppat.1007949.s006.tif]

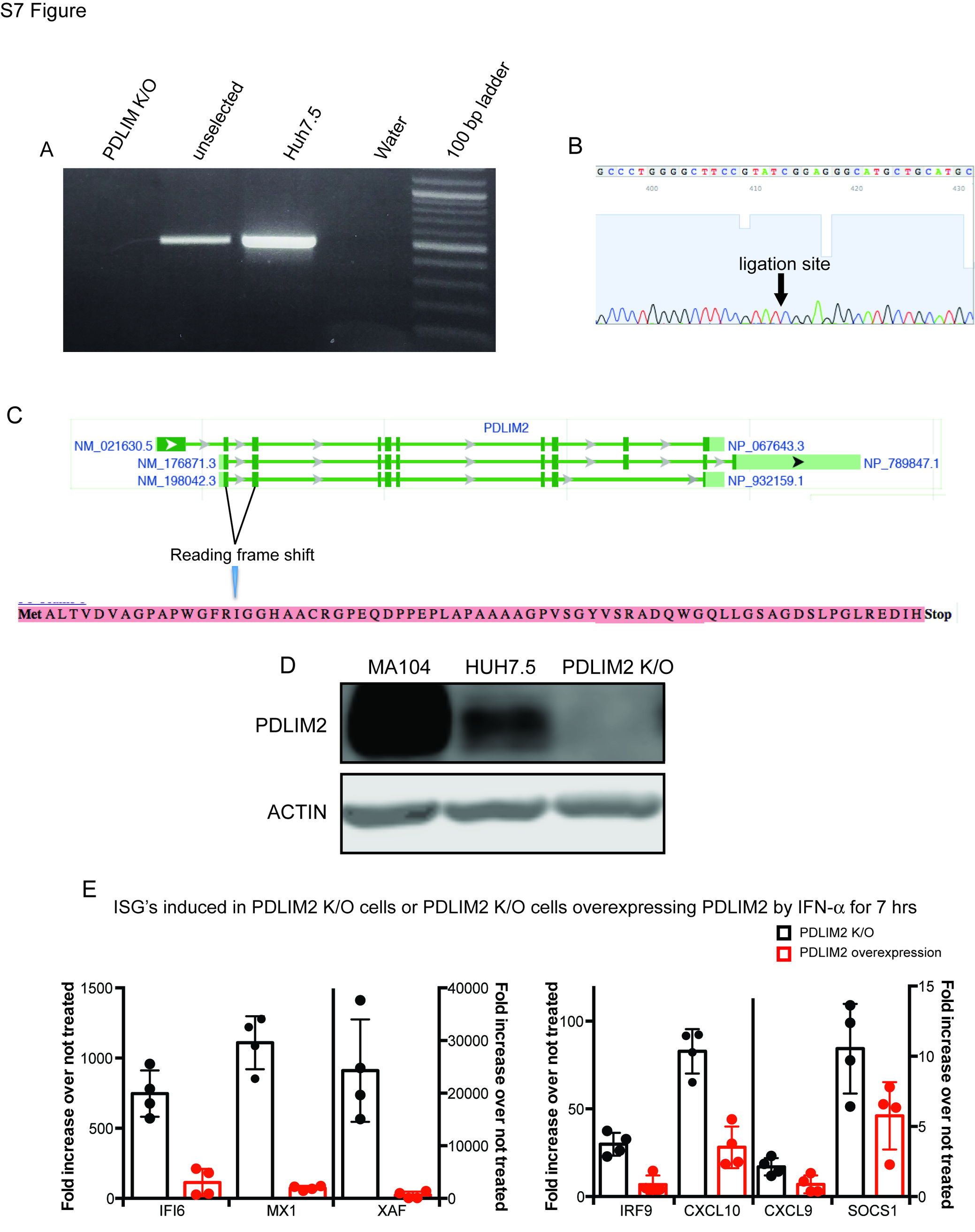

Supplement: S7 Fig — CRISPR/Cas9 was used to direct cleavage at 2 sites, the first in exon 1 and the second in exon 2 of the PDLIM2 gene as described in Materials and Methods. Blunt end ligation of DNA cleaved at both sites by cellular repair pathways yielded a deletion at the PDLIM2 locus in some cells. A clonal line of cells containing this deletion was isolated and characterized. A) PCR amplification products using primers from within the deleted region using template DNA from: parental Huh7.5 cells, an initial PDLIM2 deletion clone with some wild type Huh7.5 contamination (C4), a purified subclone of C4 designated the PDLIM K/O cell line, and a no template control. No trace of the deleted region could be detected in the purified clone. B) Sequence at the deletion breakpoint. T6914 and C7715 of the PDLIM2 gene (RefSeq NG_030435.1) have been ligated in the PDLIM2 K/O derivative of Huh7.5 with the deletion of the intervening 800 bp on both chromosomes. C) Diagram of PDLIM2 exons and the position of the deletion between the first and second exons, and the resulting presumed protein sequence. D) Western blot of MA104, Huh7.5, and PDLIM2 K/O cells with antibodies specific to PDLIM2. E) Interferon response in PDLIM K/O cells transfected with either a vector expressing double GFP protein (black), or PDLIM2 (red). PDLIM K/O cells were transfected 24h prior to treatment for an additional 7h with IFNα, lysed in Qiazol, RNA isolated and qRT-PCR was performed using PrimeTime qRTPCR assays (IDT) for 7 ISGs (S2 Table). (TIF) [file ppat.1007949.s007.tif]
