# Supplementary material for: HCV and flaviviruses hijack cellular mechanisms for nuclear STAT2 degradation: Up-regulation of PDLIM2 suppresses the innate immune response
Source: PLoS Pathog. 2019 Aug 2;15(8):e1007949. doi: 10.1371/journal.ppat.1007949 (PMC6677295; doi:10.1371/journal.ppat.1007949)
Supplement: S1 Table — Huh7.5 cells either uninfected or infected with HCV JFH-1 for 2, 4 or 7 days in duplicate wells (A and B) were lysed in Qiazol, RNA isolated, and qRT-PCR performed using a custom TaqMan OpenArray. The results for 41 genes, predominantly ISGs, are shown. The CT values were standardized to HPRT mRNA levels and the average of the duplicate uninfected sample was used to determine the fold increase in ISG gene expression after infection. (PDF) [file ppat.1007949.s008.pdf]

S1 Table.

Gene:		TRIM21	IRF7	IRF9	OAS3	MX1	ISG20	IFI6	TICAM1	VISA	IRF3	1FI44	IFIT2	IFIT3	CXCL9	RELA	CXCL10	CXCL11	ICAM1
Day2	UN	1.00 (0.94-1.07)	1.00 (0.94-1.07)	1.00 (0.92-1.09)	1.00 (0.92-1.08)	1.00 (0.93-1.08)	1.00 (0.89-1.13)	1.00 (0.91-1.10)	1.00 (0.85-1.18)	1.00 (0.94-1.07)	1.00 (0.81-1.23)	1.00 (0.94-1.07)	1.00 (0.94-1.07)	1.00 (0.94-1.07)	1.00 (0.60-1.67)	1.00 (0.91-1.09)	1.00 (0.92-1.08)	1.00 (0.66-1.51)	1.00 (0.94-1.07)
	HCV A	1.39 (1.19-1.62)	ND	1.35 (1.06-1.74)	1.03 (0.84-1.26)	0.95 (0.88-1.03)	1.37 (1.27-1.47)	0.85 (0.80-0.91)	1.30 (1.19-1.42)	1.22 (1.14-1.29)	0.71 (0.64-0.80)	4.75 (4.43-5.09)	2.61 (2.30-2.96)	1.30 (0.98-1.72)	0.51 (0.32-0.80)	1.27 (1.19-1.35)	2.03 (1.86-2.21)	0.62 (0.40-0.97)	1.33 (1.25-1.41)
	HCV B	1.17 (0.94-1.46)	ND	1.16 (1.09-1.23)	1.03 (0.90-1.18)	1.01 (0.95-1.08)	1.58 (1.41-1.77)	0.92 (0.87-0.98)	1.20 (1.13-1.27)	1.17 (1.09-1.25)	0.77 (0.69-0.85)	5.92 (5.11-6.85)	2.32 (2.09-2.57)	1.13 (1.06-1.20)	0.26 (0.19-0.35)	1.14 (1.04-1.24)	1.25 (1.13-1.38)	0.94 (0.82-1.08)	1.18 (1.11-1.25)
Day4	UN	1.00 (0.93-1.08)	1.00 (0.97-1.04)	1.00 (0.93-1.07)	1.00 (0.68-1.46)	1.00 (0.97-1.04)	1.00 (0.89-1.13)	1.00 (0.89-1.12)	1.00 (0.87-1.15)	1.00 (0.94-1.07)	1.00 (0.96-1.05)	1.00 (0.97-1.04)	1.00 (0.94-1.07)	1.00 (0.94-1.06)	1.00 (0.60-1.67)	1.00 (0.96-1.04)	1.00 (0.77-1.30)	1.00 (0.75-1.33)	1.00 (0.96-1.04)
	HCV A	4.74 (4.43-5.06)	1.93 (1.93-1.93)	8.44 (8.06-8.84)	9.20 (8.83-9.59)	10.98 (10.6-11.4)	13.26 (13.0-13.6)	3.23 (2.93-3.56)	6.10 (5.44-6.83)	2.47 (2.44-2.51)	2.02 (1.85-2.21)	464.8 (438-493)	98.62 (97.4-99.9)	15.12 (15.1-15.2)	7.34 (6.53-8.25)	2.92 (2.86-2.98)	22.05 (19.6-24.9)	9.96 (8.79-11.3)	8.53 (8.09-9.01)
	HCV B	4.00 (3.81-4.19)	5.11 (4.52-5.78)	7.47 (7.01-7.96)	7.78 (5.79-10.5)	10.01 (9.24-10.9)	13.85 (13.3-14.5)	2.97 (2.38-3.70)	5.15 (4.74-5.60)	2.03 (1.93-2.13)	1.68 (1.54-1.83)	398.2 (378-419)	100.6 (97.9-106)	17.14 (14.2-20.8)	7.72 (5.39-11.0)	2.65 (2.46-2.87)	17.13 (15.8-18.6)	13.01 (11.6-14.6)	6.73 (6.45-7.03)
Day7	UN	1.00 (0.94-1.07)	1.00 (0.87-1.15)	1.00 (0.94-1.06)	1.00 (0.61-1.64)	1.00 (0.94-1.06)	1.00 (0.97-1.03)	1.00 (0.85-1.18)	1.00 (0.94-1.07)	1.00 (0.90-1.11)	1.00 (0.90-1.11)	1.00 (0.98-1.02)	1.00 (0.89-1.13)	1.00 (0.78-1.28)	1.00 (0.46-2.20)	1.00 (0.98-1.02)	1.00 (0.94-1.07)	1.00 (0.98-1.02)	1.00 (0.94-1.06)
	HCV A	7.04 (6.52-7.61)	1.35 (1.06-1.74)	9.24 (8.48-10.1)	29.68 (27.0-32.6)	24.34 (22.4-26.5)	11.70 (10.8-12.6)	9.32 (8.07-10.8)	12.21 (11.3-13.2)	3.92 (3.61-4.25)	2.62 (2.41-2.85)	361.7 (319-410)	33.03 (29.6-36.9)	74.89 (68.5-81.9)	10.9 (8.72-13.6)	4.20 (3.86-4.57)	54.12 (45.3-64.7)	1.24 (1.14-1.34)	5.54 (5.12-5.98)
	HCV B	6.38 (6.20-6.57)	3.00 (0.94-9.32)	7.48 (6.95-8.04)	31.31 (28.9-33.9)	23.14 (22.5-23.9)	12.62 (11.5-13.9)	9.02 (8.55-9.52)	9.33 (8.58-10.2)	3.54 (3.40-3.70)	1.83 (1.75-1.92)	298.5 (270-330)	29.79 (29.2-31.5)	56.42 (44.1-72.2)	9.75 (7.00-13.6)	3.61 (3.34-3.90)	43.27 (41.2-45.5)	3.46 (2.43-4.93)	5.00 (4.80-5.20)

Gene:		IL18	IL15	IFNA2	IFNB1	IFNG	IFIH1	STAT1	STAT2	SOCS1	SOCS3	HLA-A	HLA-C	MICA	ULBP2	PDLIM2	ISG15	USP18	NLRX1
Day2	UN	1.00 (0.93-1.07)	1.00 (0.90-1.11)	1.00 (0.72-1.39)	1.00 (0.94-1.07)	1.00 (0.94-1.07)	1.00 (0.93-1.08)	1.00 (0.94-1.07)	1.00 (0.92-1.09)	1.00 (0.92-1.09)	1.00 (0.88-1.14)	1.00 (0.94-1.07)	1.00 (0.90-1.11)	1.00 (0.94-1.07)	1.00 (0.91-1.10)	1.00 (0.93-1.07)	1.00 (0.93-1.07)	1.00 (0.93-1.07)	1.00 (0.92-1.09)
	HCV A	1.36 (1.24-1.49)	1.14 (1.01-1.28)	0.52 (0.49-0.55)	0.75 (0.17-3.36)	ND	2.62 (2.45-2.80)	1.22 (1.14-1.30)	1.16 (1.08-1.24)	1.16 (1.06-1.27)	0.96 (0.90-1.03)	1.09 (1.03-1.16)	1.64 (1.48-1.81)	0.42 (0.06-2.75)	1.38 (1.20-1.59)	1.56 (1.41-1.73)	1.07 (1.00-1.15)	1.12 (1.03-1.22)	1.27 (1.11-1.46)
	HCV B	1.51 (1.40-1.63)	1.20 (0.94-1.53)	0.75 (0.53-1.06)	3.57 (3.38-3.78)	ND	1.99 (1.23-3.22)	1.10 (1.02-1.17)	1.19 (1.12-1.26)	1.22 (1.15-1.29)	0.89 (0.75-1.06)	1.14 (1.07-1.21)	1.25 (0.94-1.65)	0.11 (0.04-0.28)	1.43 (1.28-1.60)	0.93 (0.84-1.04)	1.08 (0.90-1.29)	1.17 (1.10-1.23)	1.10 (0.92-1.32)
Day4	UN	1.00 (0.95-1.06)	1.00 (0.93-1.08)	1.00 (0.94-1.06)	1.00 (0.27-3.67)	1.00 (0.94-1.07)	1.00 (0.50-1.99)	1.00 (0.95-1.06)	1.00 (0.96-1.04)	1.00 (0.95-1.05)	1.00 (0.88-1.14)	1.00 (0.97-1.04)	1.00 (0.90-1.11)	1.00 (0.19-5.18)	1.00 (0.97-1.04)	1.00 (0.96-1.05)	1.00 (0.94-1.07)	1.00 (0.91-1.09)	1.00 (0.97-1.04)
	HCV A	4.96 (4.94-4.98)	6.77 (6.26-7.31)	5.96 (5.47-6.48)	6.24 (5.21-7.48)	ND	177.9 (170-187)	2.38 (2.19-2.59)	2.83 (2.72-2.94)	0.72 (0.65-0.79)	14.69 (14.4-15.0)	3.10 (3.07-3.14)	2.56 (2.32-2.82)	40.85 (24.3-68.7)	6.02 (5.46-6.63)	14.52 (14.3-14.7)	39.09 (33.8-48.3)	1.73 (1.67-1.80)	4.47 (4.16-4.81)
	HCV B	5.38 (5.08-5.69)	5.30 (4.8-5.9)	2.13 (1.09-4.13)	5.45 (4.10-7.25)	24.02 (20.1-28.7)	184.5 (177-192)	2.03 (1.95-2.12)	2.54 (2.30-2.80)	0.46 (0.42-0.50)	13.12 (12.1-14.3)	2.86 (2.74-2.99)	3.18 (2.68-3.77)	2.18 (0.51-9.28)	4.26 (3.92-4.63)	12.53 (10.6-14.9)	40.87 (37.6-44.4)	1.69 (1.60-1.78)	3.31 (3.02-3.64)
Day7	UN	1.00 (0.92-1.08)	1.00 (0.94-1.07)	1.00 (0.91-1.10)	1.00 (0.68-1.46)	1.00 (0.94-1.07)	1.00 (0.85-1.18)	1.00 (0.90-1.12)	1.00 (0.95-1.05)	1.00 (0.89-1.12)	1.00 (0.82-1.23)	1.00 (0.97-1.04)	1.00 (0.96-1.04)	1.00 (0.95-1.05)	1.00 (0.98-1.02)	1.00 (0.95-1.06)	1.00 (0.87-1.15)	1.00 (0.92-1.09)	1.00 (0.91-1.10)
	HCV A	17.29 (15.9-18.8)	5.84 (5.00-6.82)	20.63 (18.7-22.8)	13.63 (10.1-18.5)	ND	77.05 (69.8-85.0)	3.65 (3.36-3.96)	3.72 (3.29-4.20)	0.94 (0.83-1.06)	11.23 (10.4-12.2)	3.70 (3.42-4.00)	4.16 (3.64-4.74)	71.71 (51.5-100)	2.77 (2.47-3.11)	8.07 (7.15-9.11)	34.59 (26.7-44.9)	3.86 (3.56-4.19)	6.91 (5.77-8.27)
	HCV B	13.74 (12.5-15.1)	5.20 (5.01-5.40)	7.14 (3.58-14.2)	6.38 (5.39-7.55)	16.15 (15.7-16.6)	93.15 (90.6-95.7)	3.33 (3.20-3.46)	3.36 (3.23-3.49)	0.94 (0.84-1.05)	8.61 (8.36-8.86)	3.79 (3.65-3.95)	3.86 (3.54-4.21)	58.67 (51.3-67.2)	2.66 (2.25-3.14)	5.61 (5.45-5.78)	38.84 (30.7-49.1)	3.85 (3.52-4.20)	4.61 (4.40-4.83)

Gene:		OTUD5	XAF-1	PCNA	HSPA5	ALB
Day2	UN	1.00 (0.94-1.07)	1.00 (0.94-1.07)	1.00 (0.93-1.07)	1.00 (0.94-1.07)	1.00 (0.90-1.11)
	HCV A	1.12 (1.05-1.19)	1.03 (0.97-1.10)	0.93 (0.87-0.99)	1.74 (1.62-1.86)	1.24 (1.15-1.33)
	HCV B	1.07 (1.01-1.13)	ND	0.99 (0.93-1.04)	1.50 (1.41-1.58)	1.21 (1.00-1.46)
Day4	UN	1.00 (0.91-1.10)	1.00 (0.97-1.04)	1.00 (0.94-1.06)	1.00 (0.95-1.05)	1.00 (0.94-1.06)
	HCV A	2.61 (2.59-2.62)	ND	0.63 (0.59-0.67)	3.50 (3.41-3.58)	1.72 (1.70-1.73)
	HCV B	2.60 (2.45-2.77)	4.59 (3.66-5.75)	0.66 (0.63-0.69)	3.41 (3.26-3.56)	1.47 (1.37-1.57)
Day7	UN	1.00 (0.96-1.04)	1.00 (0.98-1.02)	1.00 (0.96-1.04)	1.00 (0.98-1.02)	1.00 (0.93-1.08)
	HCV A	2.39 (2.08-2.74)	2.71 (2.45-2.99)	2.36 (2.14-2.59)	5.54 (5.08-6.04)	1.89 (1.75-2.04)
	HCV B	2.37 (2.27-2.47)	1.51 (1.47-1.55)	1.99 (1.85-2.13)	5.82 (5.46-6.21)	1.80 (1.69-1.91)

0.1-0.3	0-2.5
0.3-0.5	2.5-5
0.5-0.7	5-7.5
0.7-0.9	7.5-10
	10+

S1 Table. **Induction of interferon response genes by HCV infection**. Huh7.5 cells were infected with 3GE of HCV strain JFH-1, for 2, 4 or 7 days in duplicate wells (A and B). RNA was isolated by Trizol extraction and cDNA made using random primers and MMLV reverse transcriptase. Quantitative PCR was done using a TaqMan OpenArray with 300ng of cDNA on an ABI 7900HT. Samples were normalized to HPRT levels and fold change compared to uninfected is displayed. Two experiments (HCV A and B) are shown.
