# Supplementary material for: HCV and flaviviruses hijack cellular mechanisms for nuclear STAT2 degradation: Up-regulation of PDLIM2 suppresses the innate immune response
Source: PLoS Pathog. 2019 Aug 2;15(8):e1007949. doi: 10.1371/journal.ppat.1007949 (PMC6677295; doi:10.1371/journal.ppat.1007949)
Supplement: S2 Table — The genes and assay numbers from the Gene Expression MicroFluidics Card with a custom Human ISG array from Applied Biosystems are shown. (PDF) [file ppat.1007949.s009.pdf]

S2 Table

Assay numbers for TaqMan, OpenArray custom PCR assay.

GAPDH-Hs99999905_m1	IRF3-Hs00155574_m1
ALB-Hs00910225_m1	IRF7-Hs00185375_m1
CCL8-Hs99999026_m1	IRF9-Hs00196051_m1
CLEC2D-Hs00203174_m1	ISG15-Hs00192713_m1
CXCL10-Hs00171042_m1	ISG20-Hs00158122_m1
CXCL11-Hs00171138_m1	MICA-Hs00741286_m1
CXCL9-Hs00171065_m1	MX1-Hs00182073_m1
HLA-A;HLA-A29.1-Hs01058806_g1	NLRX1-Hs01123237_m1
HLA-C-Hs03044135_m1	OAS3-Hs00196324_m1
HLA-DRA-Hs00219575_m1	OASL-Hs00388714_m1
HLA-G-Hs00365950_g1	OTUD5-Hs01012043_m1
HPRT1-Hs99999909_m1	PCNA-Hs00952870_g1
HSPA5-Hs00946084_g1	PDLIM2-Hs00917389_m1
ICAM1-Hs00164932_m1	RELA-Hs01042010_m1
IFI44-Hs00197427_m1	SOCS1-Hs00864158_g1
IFI6-Hs00242571_m1	SOCS3-Hs02330328_s1
IFIH1-Hs01070332_m1	STAT1-Hs01013989_m1
IFIT2-Hs00533665_m1	STAT2-Hs01013123_m1
IFIT3-Hs00155468_m1	TICAM1-Hs01090712_m1
IFNA2-Hs02621172_s1	TRIM21-Hs00172616_m1
IFNB1-Hs01077958_s1	ULBP2-Hs00607609_mH
IFNG-Hs00174143_m1	USP18-Hs00276441_m1
IL15-Hs99999039_m1	VISA-Hs00325038_m1
IL18-Hs99999040_m1	XAF1-Hs00213882_m1

Primers and probes:	Forward (5'-3')	Probe (5'FAM--3'TAMARA)	Reverse
HCV:	TCTGCGGAACCGGTGAGTA	CACGGTCTACGAGACCTCCCGGGGCAC	GTGTTTCTTTTGGTTTTTCTTTGAGGTTTAG[illegible]
HAV:	GGTAGGCTACGGGTGAAAC	CTTAGGCTAATACTTCTATGAAGAGATGC	AACAACTCACCAATATCCGC
ZIKV:	CCGCTGCCCAACACAAG	AGCCTACCTTGACAAGCAGTCAGACACTCAA	CCACTAACGTTCTTTTGCAGACAT
CXCL9	AGGAACCCCAGTAGTGAGAAAGG	CCTGCATCAGCACCAACCAAGGGA	GGTCTTTCAAGGATTGTAGGTGGAT
CXCL10	TGAAAAAGAAGGGTGAGAAGAGATG	CTGAATCCAGAATCGAAGGCCATCAAGA	CCTTTCCTTGCTAACTGCTTTCAG
IFI6	AAGGCCCTGACCTTCAT	AGGAGGACTCGCAGTCGCC	ATTCAGGATCGCAGACCA
IRF9	GCCCTACAAGGTGTATCAGTTGCT	CCACCAGGAATCGTCTCTGGCCA	TCGCTTTGATGGTACTTTCTGAGT
MX1	AAGGAATGGGAATCAGTCATGAG	CACCCTGGAGATCAGCTCCCGA	TCTATTAGAGTCAGATCCGGGACAT
OAS1	TGTGTGTCCAAGGTGGTAAAGG	CCTCAGGCAAGGGCACCACCCT	CAACCAGGTCAGCGTCAGATC

G
